# ISOpureR: an R implementation of a computational purification algorithm of mixed tumour profiles

**DOI:** 10.1186/s12859-015-0597-x

**Published:** 2015-05-14

**Authors:** Catalina V Anghel, Gerald Quon, Syed Haider, Francis Nguyen, Amit G Deshwar, Quaid D Morris, Paul C Boutros

**Affiliations:** 1 Informatics and Biocomputing Program, Ontario Institute for Cancer Research, 661 University Avenue, Toronto, Suite 510, M5G 0A3 ON Canada; 2 Department of Computer Science, University of Toronto, 10 King’s College Road, Room 3303, M5S 3G4, Toronto, ON Canada; 3 Department of Oncology, University of Oxford, Old Road Campus Research Building, Roosevelt Drive, Oxford, OX3 7DQ United Kingdom; 4 Edward S. Rogers Sr. Department of Electrical and Computer Engineering, University of Toronto, 10 King’s College, Room SFB540, Toronto, M5S 3G4 ON Canada; 5 Department of Molecular Genetics, University of Toronto, 1 King’s College Circle, Room 4396, Toronto, M4S 1A8 ON Canada; 6 The Donnelly Centre, 160 College Street, Room 230, Toronto, M5S 3E1 ON Canada; 7 Department of Medical Biophysics, University of Toronto, 101 College Street, Toronto, M5G 1L7 ON Canada; 8 Department of Pharmacology and Toxicology, University of Toronto, 1 King’s College Circle, Toronto, M5S 1A8 ON Canada

**Keywords:** Tumour heterogeneity, mRNA abundance profile, Deconvolution

## Abstract

**Background:**

Tumour samples containing distinct sub-populations of cancer and normal cells present challenges in the development of reproducible biomarkers, as these biomarkers are based on bulk signals from mixed tumour profiles. ISOpure is the only mRNA computational purification method to date that does not require a paired tumour-normal sample, provides a personalized cancer profile for each patient, and has been tested on clinical data. Replacing mixed tumour profiles with ISOpure-preprocessed cancer profiles led to better prognostic gene signatures for lung and prostate cancer.

**Results:**

To simplify the integration of ISOpure into standard R-based bioinformatics analysis pipelines, the algorithm has been implemented as an R package. The *ISOpureR* package performs analogously to the original code in estimating the fraction of cancer cells and the patient cancer mRNA abundance profile from tumour samples in four cancer datasets.

**Conclusions:**

The *ISOpureR* package estimates the fraction of cancer cells and personalized patient cancer mRNA abundance profile from a mixed tumour profile. This open-source R implementation enables integration into existing computational pipelines, as well as easy testing, modification and extension of the model.

**Electronic supplementary material:**

The online version of this article (doi:10.1186/s12859-015-0597-x) contains supplementary material, which is available to authorized users.

## Background

Tumour heterogeneity provides both challenges and opportunities in the development of cancer biomarkers. Tumours are mixed populations of multiple cell-types. Currently, the molecular profiles of interest – those of cancer cells or of distinct sub-populations of cancer cells – are blurred by the mixed signal from all cell types in a sample [1,2]. However, characterizing the heterogeneity of a patient’s tumour by identifying the sub-populations present, along with their proportions and molecular profiles, would provide a personalized cancer “fingerprint” that captures both cell-centred and whole-system information, opening up opportunities for targeted treatment [3,4]. The methods described in this article apply to mRNA expression data rather than to to DNA data (point mutations and copy number changes), which require other approaches [5-8].

As a first step, it is important to consider the two-population problem of normal and cancer cells. Even small fractions of contaminating normal cells can introduce noise in gene signatures [9,10], motivating the search for methods to deconvolve a mixed tumour profile by estimating the fraction of cancer cells and providing a personalized, purified mRNA abundance profile of the cancer cells.

Physical approaches for sample separation, such as laser capture micro-dissection [11] are costly, time-intensive, not always available and may degrade samples. Therefore, computational approaches to purification of tumour molecular profiles have become increasingly important. Table 1 and Additional file 1 summarize the different methods currently available for deconvolving mRNA data.

**Table 1:** **An overview of computational deconvolution algorithms for RNA profiles**

**Method**	**Ref.**	**Input**	**Output**			**Clinical data?**			**Availability**			
			**Prop.**	**Expr.**	**Individual**	**Cancer**	**Normal blood**	**Other**	**R**	**CellMix**	**MATLAB**	**Other**
					**profile**							
ISOpure (Quon)	[33]	tumour & unmatched normal	$\checkmark $	$\checkmark $	$\checkmark $	$\checkmark $			$\checkmark $		$\checkmark $	
DeMix (Ahn)	[32]	tumour & unmatched normal	$\checkmark $	$\checkmark $	$\checkmark $				$\checkmark $			
Clarke	[30]	paired mixed & pure profiles	$\checkmark $		$\checkmark $				$\checkmark $			
Gosink	[31]	mixed profiles and known profile of one constituent	$\checkmark $		$\checkmark $							
DeconRNASeq (Gong)	[18]	profiles of constituents	$\checkmark $						$\checkmark $			
Gong	[19]	cell-type specific gene signatures	$\checkmark $							$\checkmark $		
Abbas	[20]	cell-type specific gene signatures	$\checkmark $				$\checkmark $			$\checkmark $		
Wang M.	[21]	cell-type specific gene signatures	$\checkmark $									
Lu	[22]	cell-type specific gene signatures	$\checkmark $									*
PERT (Qiao)	[46]	reference profiles of constituents	$\checkmark $	†	†		$\checkmark $					$\checkmark $
ESTIMATE (Yoshihara)	[47]	prior data used to derive cell-type specific gene signatures	$\checkmark $			$\checkmark $			$\checkmark $			
DSection (Erkkilä)	[12]	prior knowledge of proportions	†	$\checkmark $						$\checkmark $	$\checkmark $	
csSAM (Shen-Orr)	[13]	proportions of constituents		$\checkmark $			$\checkmark $		$\checkmark $	$\checkmark $		
Bar-Joseph	[14]	proportions of consitutents, one expression profile		$\checkmark $		$\checkmark $		$\checkmark $				
Ghosh	[16]	proportions, tumour & unmatched normal		$\checkmark $		$\checkmark $			*			
Stuart	[17]	proportions of constitutents		$\checkmark $		$\checkmark $						
TEMT (Li)	[48]	prior knowledge of proportions, paired mixed-pure profiles			$\checkmark $							$\checkmark $
DSA (Zhong)	[23]	cell markers	$\checkmark $	$\checkmark $		$\checkmark $			$\checkmark $	$\checkmark $		
ssNMF (Gaujoux)	[25]	cell markers	$\checkmark $	$\checkmark $			$\checkmark $			$\checkmark $		
PSEA (Kuhn)	[24]	cell markers	$\checkmark $	$\checkmark $				$\checkmark $	$\checkmark $			
deconf (Repsilber)	[26]	cell markers	$\checkmark $	$\checkmark $			$\checkmark $		$\checkmark $	$\checkmark $		
Tolliver	[49]	tumour profile, number of constituents	$\checkmark $	$\checkmark $		$\checkmark $						
Roy	[50]	prior estimate of number of constituents	$\checkmark $	$\checkmark $								
Lähdesmäki	[15]	mixed expression profiles	†	$\checkmark $								
Venet	[27]	mixed expression profiles, number of constituents	$\checkmark $	$\checkmark $		$\checkmark $						
UNDO (Wang N.)	[51]	mixed expression profiles	$\checkmark $	$\checkmark $		$\checkmark $			$\checkmark $			

Most of the algorithms are applied to microarray mRNA abundance data, although TEMP and ESTIMATE use high-throughput RNA-Seq data and ISOpure and DeconRNASeq can be applied to both [52]. The possible outputs of the algorithms are proportions of constituent cell-types (Prop.), average expression profiles (Expr.), or patient-specific expression profiles (Individual Profile) of constituent cell-types. The two main sources of clinical data were cancer-related gene expression data (including human Hodgkin’s lymphomas) or normal blood expression data. PSEA was applied to expression data from patients with Huntington’s disease, and Bar-Joseph also studied cell cycle synchronized foreskin fibroblast cells. In terms of availability, the summary package CellMix [28] is also an R package but is listed as a separate category. The only algorithms not available for either R or MATLAB are PERT (Octave) and TEMT (Python). Algorithms which were described as using built-in MATLAB or R functions were not included, as reproducible example code is not available for them. The currently available source code is summarized in Additional file 2.

Notes:

^†^Prior information about proportions or expressions is needed, but these values are re-estimated during the execution of the algorithm. For PERT, the individual profiles are adjusted (perturbed) versions of the reference profiles.

^*^The original code for Lu (Java-based) [22] and Ghosh [16] is no longer available.

Classical methods of profile deconvolution assume that a mixed profile is a linear combination of a predetermined number of pure constituent profiles. Written in matrix form, the measured, mixed profiles *B* are a product of *A*, a matrix of gene expression profiles of each constituent, weighted by the fractions *X* of each cell type in the mixture:

(1) $$ AX=B.  $$

Equivalently, some algorithms start with the transpose of this equation. Different algorithms use different methods of deconvolution. Some assume that the fractions, *X*, are known [12-17]. Others use gene expression profiles [18], signatures [19-22] or markers [23-26] of the constituent profiles to recover *X*, and sometimes the expression profiles of other genes in the constituents [23]. (A gene marker is a set of genes assumed to be expressed solely in one cell subtype and in no other [4,27,28].) These approaches are limited in uncovering patient-specific variation in cancer, as they assume that all tumour profiles are mixtures of a small number of the same constituents. In addition, the expression data may be log-transformed [13,16], leading to a possible bias in the reconstruction of mixed tissue samples from constituent profiles [29].

Another approach to estimate both fraction of cancer content as well as patient-specific cancer profiles requires a matched normal profile for each patient [30,31]. In this case the normal profile is “electronically subtracted” from the bulk tumour profile. However, a matched normal profile may not always be available in existing datasets and may be difficult to obtain clinically. Furthermore, due to the biological variability of normal tissue, the provided normal profile may not match that of the tissue in the tumour sample.

Two algorithms, DeMix [32] and ISOpure [33] present statistical approaches for deconvolution of mixed tumour profiles given a set of unmatched normal samples. Cancer biomarkers generated from prostate and non-small cell lung cancer data purified using ISOpure were more effective at predicting survival relative to those generated using unpurified profiles [33].

### Overview of ISOpure

In the following, we will use the term ‘ISOpure’ to refer to the algorithm in general, and *ISOpureR* to refer to the R package implementation. The next two sections will describe the statistical model and the algorithm in more detail, but we begin by providing a brief overview and example.

For the applications discussed here, the ISOpure algorithm is applied to microarray mRNA abundance data. The inputs to the model must be normalized (but not log-transformed) expression profiles. The following two sets of inputs are required: tumour mRNA abundance profiles, of the same cancer sub-type; and normal (*i.e.* healthy) mRNA abundance profiles, from the same tissue as the tumour.

The algorithm runs in two steps.

1. **Cancer Profile Estimation (CPE).** This step estimates and outputs an average cancer profile as well as a fraction of cancer in each tumour sample.
2. **Patient Profile Estimation (PPE).** This step estimates and outputs a cancer profile for each patient.These profiles are all similar to the average cancer profile, but contain patient-specific variations. The estimated cancer fraction for each tumour sample is fixed at the value calculated in the CPE step.

To run these two steps using *ISOpureR*, it suffices to apply the two functions ISOpure.step1.CPE and ISOpure.step2.PPE. The input expression data should be in matrix form, with samples along the columns and transcripts/features along the rows.



The vector ISOpureS1model$alphapurities produced by the first step contains the proportion of cancer for each patient. The matrix ISOpureS2 model $cc_cancerprofiles produced by the second step contains the patient-specific profiles, rescaled to be of the same scale as the tumour expression data. That is, these profiles are estimated as probabilities within the algorithm, but are scaled to represent microarray signal intensity data. A detailed example is given in Section 3 of the *ISOpureR* package vignette, included as Additional file 2.

**Figure 1:**
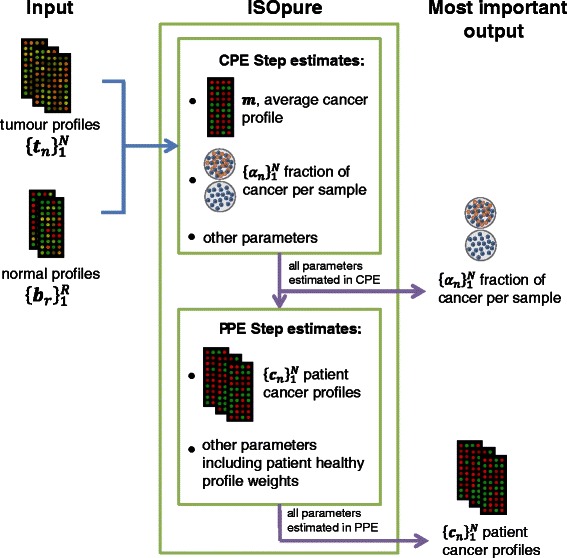
ISOpure workflow. An overview of the ISOpure algorithm, illustrating the inputs and the most important outputs of the Cancer Profile Estimation (CPE) and the Patient Profile Estimation (PPE) steps. The CPE step estimates an average cancer profile over all patients and the proportion of cancer in each tumour, as well as the patient healthy profiles (as weights of input profiles). The healthy profile weights are re-estimated in the PPE step. This second step estimates the purified cancer profile for each patient. All estimated parameters from the CPE and PPE steps are output by the *ISOpureR* functions ISOpure.step1.CPE and ISOpure.step2.PPE.

In the statistical and algorithm descriptions of ISOpure, the notation **t**_*n*_ describes the matrix of tumour profiles, where the index *n* denotes the *n*-th patient. The fractions *α*_*n*_ represent the cancer fractions. The **b**_*r*_ and **c**_*n*_ represent normal and purified cancer profiles, but they will be interpreted as probabilities, as described in more detail below. An overview of the algorithm workflow is given in Figure 1.

While we focus on microarray mRNA abundance data, ISOpure is generic when it comes to different species of RNA. For example, it is able to deconvolve both mRNA (non-coding RNAs inclusive) and microRNAs. This is true for both microarray as well as next-generation sequencing (RNA-Seq) data. Input data matrices can represent genes, isoforms, exons or microRNAs of samples. The data matrices should represent approximate counts of molecules (normalised but not log converted) for a given RNA profile, *e.g.* gene level mRNA. However, the input should not contain a mixture of mRNA and microRNA data. ISOpure has been applied to the RNA-Seq The Cancer Genome Atlas (TCGA) PRAD dataset as part of that project (manuscript in preparation), helping to demonstrate its wider applicability.

### The ISOpure statistical model

A more complete explanation of the statistical model is given in the original article, [33], as well as in the *ISOpureR* package vignette (Additional file 2). As noted, ISOpure addresses the two-population problem, assuming that a patient’s particular tumour mRNA abundance profile **t**_*n*_ can be decomposed into its cancer and healthy profile components. For patient *n*,

(2) $$ \mathbf{t}_{n} = \alpha_{n} \mathbf{c}_{n} + (1-\alpha_{n}) \mathbf{h}_{n} + \mathbf{e}_{n}.  $$

Here, **c**_*n*_ is the personalized cancer profile, **h**_*n*_ the profile of the patient’s healthy tissue, *α*_*n*_ the fraction of cancer cells (0≤*α*_*n*_≤1) and **e**_*n*_ the reconstruction error.

This linear system of equations (for patients 1 to *N*) is underdetermined, as the only known values are the **t**_*n*_’s. To reduce the number of parameters to be estimated, as well as to prevent overfitting, ISOpure employs two regularization techniques. First, the patient-specific healthy profile, **h**_*n*_ is assumed to be a convex combination of a reference set of known healthy profiles, **b**_1_,…,**b**_*n*_:

(3) $$ \mathbf{t}_{n} = \alpha_{n} \mathbf{c}_{n} + \sum_{r=1}^{R} \theta_{n,r} \mathbf{b}_{r} + \, \mathbf{e}_{n}  $$

where

(4) $$ \alpha_{n} + \sum_{r=1}^{R} \theta_{n,r} = 1.  $$

This assumption is convenient for data availability (a paired normal sample is not always available in archived datasets), but is also motivated by biology; even a paired healthy sample may not correspond exactly with the healthy portion of the tumour sample, and may contain noise. The second regularization assumption is that the cancer profiles, **c**_*n*_ cluster near an estimated reference cancer profile **m**, an assumption that is more accurate when the cancers are all of the same subtype [34,35].

For the statistical model, the cancer profiles, **m** and **c**_*n*_, and the healthy profiles, **b**_*r*_, are transformed into probability distributions. Thus,

(5) $$ \hat{\mathbf{x}}_{n} = \alpha_{n} \mathbf{c}_{n} + \sum_{r=1}^{R} {\theta_{n,r} \mathbf{b}_{r}}   $$

becomes a probability vector. The tumour sample **t**_*n*_ is discretized to **x**_*n*_, and considered to be a sample from the multinomial distribution with probability vector $\hat {\mathbf {x}}_{n}$.

The following equations describe the full statistical model, which is also illustrated in the Bayesian network diagram in Additional file 3. To simplify notation, the vector ***θ***_*n*_ includes the entries *θ*_*n*,1_,*θ*_*n*,2_,…,*θ*_*n*,*R*_,*α*_*n*_.

(6) $$\begin{array}{*{20}l} \mathbf{B} &=\, [\mathbf{b}_{1}, \ldots, \mathbf{b}_{R}]  \end{array} $$

(7) $$\begin{array}{*{20}l} \hat{\mathbf{x}}_{n} &=\, [\mathbf{B} \, \mathbf{c}_{n}] \boldsymbol{\theta}_{n}  \end{array} $$

(8) $$\begin{array}{*{20}l} p\left(\mathbf{x}_{n}|\mathbf{B}, \boldsymbol{\theta}_{n}, \mathbf{c}_{n} \right) &= \text{Multinomial}\, (\mathbf{x}_{n}|\hat{\mathbf{x}}_{n})  \end{array} $$

(9) $$\begin{array}{*{20}l} p\left(\boldsymbol{\theta}_{n}| \boldsymbol{\nu} \right) &= \text{Dirichlet}\left(\boldsymbol{\theta}_{n}|\boldsymbol{\nu} \right)  \end{array} $$

(10) $$\begin{array}{*{20}l} p\left(\mathbf{c}_{n}|k_{n},\mathbf{m} \right) &= \text{Dirichlet}\left(\mathbf{c}_{n}|k_{n}\mathbf{m} \right)  \end{array} $$

(11) $$\begin{array}{*{20}l} p\left(\mathbf{m}|k', \mathbf{B}, \boldsymbol{\omega} \right) &= \text{Dirichlet}\left(\mathbf{m}|k'\mathbf{B} \boldsymbol{\omega} \right)  \end{array} $$

Equation (7) is simply Equation (5) in matrix form, and Equation (8) summarizes the model described in the previous paragraphs.

The Dirichlet distributions are used for the parameters ***θ***_*n*_, **m** and **c**_*n*_, which are discrete probability distributions. The hyper-parameters ***ν***, *k*_*n*_, *k* ^′^ and ***ω*** determine the mean and the concentration of the Dirichlet distributions.

### The ISOpure algorithm

The goal of the algorithm is to maximize the complete likelihood function

(12) $$  \mathbb{L} = p\left(\mathbf{m}|k', \mathbf{B}, \boldsymbol{\omega} \right) \prod_{n=1}^{N}{p\left(\mathbf{c}_{n}|k_{n},\mathbf{m} \right)p\left(\boldsymbol{\theta}_{n}| \boldsymbol{\nu} \right) p\left(\mathbf{x}_{n}|\mathbf{B}, \boldsymbol{\theta}_{n}, \mathbf{c}_{n} \right)}.  $$

The ISOpure algorithm splits this optimization into two steps. The Bayesian diagram and the flowchart of the algorithm for each step are given in Additional file 4. ISOpure uses block coordinate descent where all variables except one (or a ‘block’ of similar variables) are fixed, and the objective is minimized with respect to the one variable (or block).

## Implementation

### Motivation and software design

The main contribution of this paper is the implementation of ISOpure in the widely-used R statistical environment. R is one of the most popular programming languages in bioinformatics, in particular for the analysis and visualization of genomic data. It is freely available under the GNU General Public Licence (GPLv2/3) and can be extended by many open-source packages.

ISOpure was originally implemented using MATLAB [33,36]. The demand for an R version of ISOpure has been motivated both by the cost-effectiveness of R, as well as by the convenience and possible customization of the algorithm in a familiar language. *ISOpureR* enables the integration of the computational purification step within existing data-analysis pipelines. The code was designed using R version 3.1.1 (64-bit) on Ubuntu 12.04.4 LTS [37].

The organization of the R implementation closely follows the original MATLAB code. While the structure of the code remains consistent, the advantage of using an R package is that help files are easily accessible, as for any package (*e.g.* typing help(package=ISOpureR) after loading the library will list all functions). The most important help file is the vignette (Additional file 2), which gives details on the algorithm, the preprocessing steps for microarray data and an extended example of running ISOpure on a computationally convenient dataset included with *ISOpureR*, with visualizations of the output. The internal test cases for the package also use this small dataset to test the log likelihood and the derivative of the log likelihood functions for each parameter.

### Comparison with the original code

Two main challenges in the translation of ISOpure from MATLAB to R were the differences in output of standard functions in the two languages, and differences in running time. Surprisingly, MATLAB and R outputs differ for two of the most basic operators: greaterthan (>) and less than (<). In MATLAB, a comparison between a number and NA or NaN returns FALSE, while in R the output is NA. (For instance, 3 < NA would return 0 in MATLAB, and NA in R.) The optimization function (ISOpure.model_optimize.cg_code.rminimize .R in *ISOpureR*, which copies the MATLAB ISOpure function) performs a line search using quadratic and cubic polynomial interpolations/extrapolations and the Wolfe-Powell stopping criteria. Intermediate iterates which fall outside the function’s domain result in infinite function or derivative values, and NaN values in the succeeding iterate. In this case, the ISOpure algorithm outputs FALSE when testing the Wolfe-Powell stopping criteria and the search continues with an adjusted search point. Thus, alternative versions of the greater and less than operators in R (*e.g.* ISOpure.util.matlab_greater_than.R) ensure the correct performance of the minimizing function in *ISOpureR*.

Another difference in MATLAB and R is the behaviour of the logarithm function for negative real values. In R, log(x) outputs NaN for negative values of x, while in MATLAB the output is a complex number. In order to avoid underflow (the numerical error resulting from computing a number too small in magnitude to store in memory), ISOpure often performs calculations in the log domain. For some of the intermediate calculations, the logarithm of a negative value is calculated (*e.g.* the logarithm of a derivative which may have negative components).

In addition to code-level differences, R and MATLAB differ in running time. MATLAB takes advantage of multi-core processing by default. While the average elapsed time for ISOpure in R was 1.73 to 2.61 times slower than the CPU-time in MATLAB, it was much slower than the elapsed time in MATLAB. To improve the runtime of *ISOpureR*, the current version (v.1.0.16) incorporates C++ code into the algorithm using RcppEigen [38], reducing the elapsed time two to three fold, from 8.9-13.4 to 3.9-4.8 times the elapsed MATLAB time. It is also useful to note that despite the slower time, for running large numbers of similar models (*e.g.* 50), the performance of *ISOpureR* was faster overall, as the jobs could be submitted simultaneously to a compute cluster, with no licence limitations.

The size of the dataset influences runtime most significantly. The running time seems to be linearly dependent on the number of transcripts/features when all other values (number of tumour samples, number of normal samples) are kept the same. Similarly, the time is also linearly dependent on the number of tumour samples and normal samples (Additional file 5).

## Results and discussion

To verify the numerical equivalence of the MATLAB and R implementations of ISOpure, their performances on four datasets were compared. Two sets were of lung adenocarcinoma from Bhattacharjee [39] and Beer [40] and two were of prostate cancer from Wallace [41] and Wang [42] (Table 2). These four datasets are among the tumour datasets purified using ISOpure in [33], chosen because the cancer types are not yet known to have established subtypes. The array data processing was detailed in [33].

**Table 2:** **An summary of the datasets used to validate** ***ISOpureR***

**Dataset**	**Ref.**	**Cancer type**	**Number of samples**		**Number of transcripts**
			**Tumour**	**Normal**	
Beer	[40]	lung adenocarcinoma	86	10	5,151
Bhattacharjee	[39]	lung adenocarcinoma	139	17	8,383
Wallace	[41]	prostate cancer	69	18	12,140
Wang	[42]	prostate cancer	109	45	18,185

Each dataset was purified using both the MATLAB and R implementations of ISOpure and we compared the resulting parameters. The algorithms were run 50 times for each dataset, with different initial conditions. To minimize differences due to random number generation implementations, the initial values of parameters were loaded from a file, and the extra optimizations of parameters ***ν***, ***ω***, and *k* [33] in the CPE step, which included some random initializations, were omitted. The results of these models are very similar to results generated by the full, randomized version of *ISOpureR*; the motivation for minimizing randomness was simply to reduce differences in the performance comparisons between MATLAB and R.

**Figure 2:**
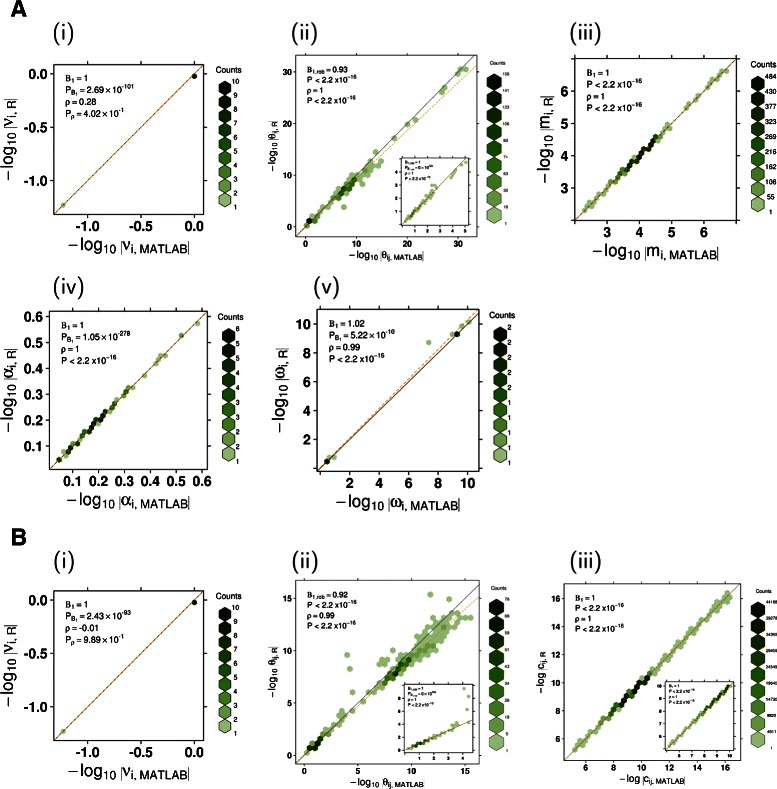
A comparison of parameters estimated by the MATLAB and R implementations of ISOpure for the Beer dataset. Each plot shows the entries of a parameter estimated using *ISOpureR* plotted against the corresponding entries estimated using the MATLAB code. The parameter is an average over 49 models run with different initial conditions (one MATLAB model for the Beer dataset resulted in a zero ***θ*** value, and was dropped). The line *y*=*x* is indicated in black, and the linear regression line, or robust regression line for ***θ***, is dashed orange. **(A)** Parameters from the Cancer Profile Estimation step of ISOpure: (i) ***ν***, the hyper-parameter for the Dirichlet distribution over ***θ***, (ii) ***θ***, the proportion of a patient sample from a known healthy-tissue profile, (iii) **m**, the average mRNA abundance cancer profile, (iv) *α*, the fraction of cancer cells for every patient sample, (v) ***ω*** a hyper-parameter for the Dirichlet distribution over **m**. **(B)** Parameters from the Patient Profile Estimation step of ISOpure: (i) ***ν***, the hyper-parameter for the Dirichlet distribution over ***θ***, (ii) ***θ***, the proportion of a patient sample from a known healthy-tissue profile, (iii) *c*_*n*_, the purified mRNA abundance cancer profile for each patient.

The numerical differences in the parameter estimates produced by MATLAB and R are small enough to have no biological significance. We calculated the means of the parameters ($\bar {\mathbf {x}}_{\textit {MATLAB}}$, $\bar {\mathbf {x}}_{R}$, for each of the estimated parameters such as ***ν***,***α***, etc.) from the MATLAB and R models over the 50 iterations. A comparison of the entries of these mean parameter values produced by R and MATLAB shows that the two implementations are numerically analogous (Figure 2 and Additional file 6). In particular, *β*_1_ and Spearman’s *ρ* are 1 for both the estimated fraction of cancer cells in the tumour, *α*, and for the log of the individual cancer profiles **c**_*n*_.

For the all datasets, the mean and median of the fractional difference between the entries of the parameter means are very close to zero for six of the nine parameters (Additional file 7). For instance, for both the Beer and the Bhattacharjee datasets the means and medians for the fractional difference of *α* and log**c**_*n*_ are between 10^−5^ and 10^−4^. The three parameters having larger differences, ***ω*** in the CPE step and ***θ*** in both CPE and PPE steps, contain very small entries which are not biologically significant. The entries of ***θ*** represent the weights of known normal-tissue profiles adding up to a patient’s particular normal profile; a weight of 10^−5^ essentially means that particular known normal profile is not present for that patient. When entries larger than a certain threshold, such as 10^−5^, are compared, the fractional differencedecreases.

Furthermore, the differences between the MATLAB and R algorithms are similar to the differences within each implementation, under different initial conditions (Additional file 8). In particular, smaller numbers are not as precisely predicted. Computationally, operations with smaller numbers are susceptible to floating point rounding errors; however differences in small numbers do not alter the model, as they are biologically unimportant.

Finally, the mean and median of the vector components of $\left (\bar {\mathbf {x}}_{\textit {MATLAB}}- \bar {\mathbf {x}}_{R} \right)$ are very close to 0, and are sometimes positive and sometimes negative. The R algorithm does seem to under-estimate parameters compared to MATLAB, but this bias disappears when we compare parameter values larger than 10^−5^.

## Conclusions

The first stage in the development of *ISOpureR* focused on establishing the numerical equivalence of the results produced by the R and MATLAB code. Future steps include testing backward compatibility with ISOLATE [43], the precursor to ISOpure.

The translation of MATLAB code into R code was surprisingly challenging. Debugging took five times as long as the translation and initial testing, as differences in function performance appeared only for certain input values during the execution of the full algorithm rather than in the testing of individual functions. Perhaps some of the differences in basic MATLAB and R operators mentioned can be of help for others tackling a similar project. A list of key issues encountered is in Additional file 9.

One of the recommendations for the comparison of implementations would be to eliminate all sources of randomness in the algorithms and postpone the improvement of running time only once numerical results are consistent, and consistently reproducible given different initial random seeds.

Most importantly, the contribution of the R implementation of the ISOpure algorithm is that it can now be readily integrated into existing analysis pipelines. *ISOpureR* can easily be included in benchmark comparisons of different deconvolution algorithms. A promising upcoming project is the ICGC-TCGA DREAM Somatic Mutation Calling - Tumour Heterogeneity Challenge [44], a benchmarking competition of computational methods for determining the best sub-clonal reconstruction algorithms. The collaborative-competitive framework of the DREAM projects encourages transparency in algorithm development and the availability of open-source code. The R implementation of ISOpure provides increased flexibility and ease of parallelization, so that the algorithm may be easily modified, extended and tested by thecommunity.

## Availability and Requirements

The *ISOpureR* package is submitted to the Comprehensive R Archive Network (CRAN) which maintains an active package homepage for *ISOpureR*. The version of the code at the time of publication is included in Additional file 10. The package is written an implemented in the R programming language (version ≥ 3.1.1), with some C++ code incorporated using Rcpp [45] and RcppEigen [38]. It is platform-independent, but has been primarily tested on Linux, and is available under the GLP-2 license.

## Additional files

Additional file 1
**(Table) A summary of software availability for deconvolution of mRNA abunance data. A similar table is given in [** 52 **].**

Additional file 2
**The vignette of the** ***ISOpureR*** **package, ISOpureRGuide.pdf, including an extended description of the ISOpure model and algorithm, preprocessing steps for microarray files, and examples for applying** ***ISOpureR*** **to datasets.** This file is also found within the *ISOpureR* package, in Additional file 10.

Additional file 3
**(Figure) Bayesian network model for the ISOpure statistical model.**

Additional file 4
**(Figures) Bayesian network and flowchart diagrams for the cancer profile estimation and patient profile estimation steps of the ISOpure algorithm.**

Additional file 5
**(Figures) A comparison of running time for different dataset sizes (different number of transcripts or different number of tumour samples).**

Additional file 6
**(Figures) A comparison of parameters estimated by the MATLAB and R implementations of ISOpure for the Bhattacharjee, Wallace, and Wang datasets.**

Additional file 7
**(Tables) Statistics for differences in parameters estimated by the MATLAB and R implementations of ISOpure for the Beer, Bhattacharjee, Wallace, and Wang datasets.**

Additional file 8
**(Figure) A comparison of the parameter estimation in models with different initial conditions, one programming language.**

Additional file 9
**Recommendations for translating code from MATLAB to R.**

Additional file 10
***ISOpureR*** **R package as a Linux-compatible file.**
