# Supplementary material for: ISOpureR: an R implementation of a computational purification algorithm of mixed tumour profiles
Source: BMC Bioinformatics. 2015 May 14;16:156. doi: 10.1186/s12859-015-0597-x (PMC4429941; doi:10.1186/s12859-015-0597-x)
Supplement: Additional file 1 — (Table) A summary of software availability for deconvolution of mRNA abunance data. A similar table is given in [ 52 ]. [file 12859_2015_597_MOESM1_ESM.pdf]

Table 1 The current code availability of each software (similar to Table 1 of [52]). As ISOpure and DeMix are similar, they are listed first; all other software is listed according to the year of publication. ISOpure is available in both MATLAB and R, and some methods have source code incorporated into the CellMix package, available at https://r-forge.r-project.org/scm/viewvc.php/pkg/CellMix/?root=cellmix&sortdir=down.

Method	Year published	Language(s)	Source
ISOpure (Quon)	2013	R	http://cran.r-project.org/web/packages/ISOpureR/index.html
		MATLAB	http://people.csail.mit.edu/geraldquon/software.shtml#isopure
DeMix (Ahn)	2013	R	http://odin.mdacc.tmc.edu/~wwang7/DeMix.html
Abbas	2009	R	CellMix: https://r-forge.r-project.org/scm/viewvc.php/pkg/CellMix/?root=cellmix&sortdir=down
deconf (Repsilber)	2010	R	CellMix & http://www.biomedcentral.com/1471-2105/11/27
Clarke	2010	R	www.biomed.miami.edu/site/clients/UMMM/user/65/doc/mixture_estimation.zip
csSAM (Shen-Orr)	2010	R	CellMix & http://cran.r-project.org/web/packages/csSAM/index.html
DSection (Erkkilä)	2010	R, MATLAB	CellMix & http://informatics.systemsbiology.net/DSection
Gong	2011	R	CellMix: https://r-forge.r-project.org/scm/viewvc.php/pkg/CellMix/?root=cellmix&sortdir=down
PSEA (Kuhn)	2011	R	http://www.bioconductor.org/packages/release/bioc/html/PSEA.html
ssNMF (Gaujoux)	2012	R	CellMix: https://r-forge.r-project.org/scm/viewvc.php/pkg/CellMix/?root=cellmix&sortdir=down
PERT (Qiao)	2012	Octave	http://people.csail.mit.edu/geraldquon/software.shtml#pert
DeconRNASeq (Gong)	2013	R	http://master.bioconductor.org/packages/release/bioc/html/DeconRNASeq.html
DSA (Zhong)	2013	R	CellMix & https://github.com/zhandong/DSA
TEMT (Li)	2013	Python	https://github.com/uci-cbcl/TEMT
ESTIMATE (Yoshihara)	2013	R	http://sourceforge.net/projects/estimateproject/
UNDO (Wang N.)	2014	R	http://master.bioconductor.org/packages/release/bioc/html/UNDO.html
