# Supplementary material for: ISOpureR: an R implementation of a computational purification algorithm of mixed tumour profiles
Source: BMC Bioinformatics. 2015 May 14;16:156. doi: 10.1186/s12859-015-0597-x (PMC4429941; doi:10.1186/s12859-015-0597-x)
Supplement: Additional file 2 — The vignette of the ISOpureR package, ISOpureRGuide.pdf, including an extended description of the ISOpure model and algorithm, preprocessing steps for microarray files, and examples for applying ISOpureR to datasets. This file is also found within the ISOpureR package, in Additional file 10. [file 12859_2015_597_MOESM2_ESM.pdf]

A guide to using *ISOpureR*

Catalina V. Anghel

April 22, 2015

Contents

1 Background **2**
1.1 Statistical model 2
1.2 Algorithm 3

2 Preprocessing steps for raw microarray data **5**
2.1 Load necessary libraries 5
2.2 Process .CEL files 6
2.3 Obtain tumor and normal expression data 8
2.4 Further processing 8
2.5 Save expression data 9
2.6 Run *ISOpureR* on the expression data 9

3 Applying *ISOpureR* to a small example **10**
3.1 Load and format data 10
3.2 Run the Cancer Profile Estimation 12
3.3 Run the Patient Profile Estimation 13

4 Visualize results from small example **15**
4.1 Proportion of cancer 15
4.2 Tumor and cancer profiles 17

5 Generating gene signatures **22**

1 Background

ISOpure is a statistical model for deconvolution of mRNA microarray profiles of mixed tumor samples into the constituent normal and cancer profiles, as well as estimating the proportion of cancer content. The model was developed by Quon et al. in [1] and first implemented in MATLAB. The R package *ISOpureR* keeps as close to the MATLAB implementation as possible. We will use 'ISOpure' to refer to the algorithm in general and *ISOpureR* to refer to the R package.

The full description of the model details (inputs, outputs, computation) is in [1]. A summary of the changes made for the R implementation is given in [3]. In particular, *ISOpureR* is not yet tested to be back-compatible with ISOLATE [2], the precursor to ISOpure.

1.1 Statistical model

The motivating equation for the ISOpure model is the decomposition of a mixed tumor profile, specific to a particular patient n, into its healthy and cancer components. By 'profile', we mean an mRNA abundance profile, obtained using microarrays or RNA sequencing. Therefore, given a tumor profile $\mathbf{t}_n$, we assume

$$\mathbf{t}_n = \alpha_n \mathbf{c}_n + (1 - \alpha_n)\mathbf{h}_n + \mathbf{e}_n,$$

where $\mathbf{c}_n$ is the personalized mRNA profile of the cancer, $\mathbf{h}_n$ is the profile of healthy tissue, α_n is the fraction of cancer, and $\mathbf{e}_n$ is the reconstruction error. At this point we know only $\mathbf{t}_n$ for patients 1 to N, making the system underdetermined.

Since patients do not necessarily have a matched tumor-normal sample, and the normal sample itself may contain noise or have a different composition from the healthy component inside the tumor sample, $\mathbf{h}_n$ is estimated from a known set of R reference normal samples $\{\mathbf{b}_r\}_1^R$. In general, $R < N$, where N is the total number of patients. The equation now becomes

$$\mathbf{t}_n = \alpha_n \mathbf{c}_n + \sum_{r=1}^{R} \theta_{n,r} \mathbf{b}_r + \mathbf{e}_n$$

with the constraint that

$$\alpha_n + \sum_{r=1}^{R} \theta_{n,r} = 1.$$

Note that $0 < \alpha_n < 1$ and $0 < \theta_{n,r} < 1$.

However, instead of considering $\mathbf{c}_n$ and $\mathbf{b}_r$ as profiles, we will normalize them into probability distributions. That is, divide each entry of $\mathbf{b}_r$ by the sum of its elements. Then the gth entry of $\mathbf{b}_r$ represents the probability of observing the transcript from gene g if draw a random transcript from the rth healthy cell sample. Repeat the procedure for $\mathbf{c}_n$. The probability vector

$$\hat{\mathbf{x}}_n = \alpha_n \mathbf{c}_n + \sum_{r=1}^{R} \theta_{n,r} \mathbf{b}_r \tag{1}$$

gives the probability of observing a certain transcript from the mixed tumor sample of patient n. Thus, ISOpure represents the tumor profile as a sample from a multinomial distribution. The discretized tumor profile $\mathbf{x}_n$ (obtained from rounding $\mathbf{t}_n$) represents a count vector from the corresponding multinomial distribution (with probability vector $\hat{\mathbf{x}}_n$) for that patient.

Finally, two regularization assumptions are used to avoid overfitting.

- The first, already noted, is that the healthy profile for a particular patient, $\mathbf{h}_n$, is assumed to be a convex combination of the reference healthy profiles $\{\mathbf{b}_r\}$.

- The second assumption is that the cancer profiles $\mathbf{c}_1, \mathbf{c}_2, \ldots, \mathbf{c}_N$ cluster together around a reference, or average cancer profile $\mathbf{m}$, an assumption that is more accurate when the cancer profiles are of the same sub-type.

The full ISOpure model is defined as follows. To simplify notation, the the vector $\boldsymbol{\theta}_n$ is of length $R+1$ containing the entries $\theta_{n,1}, \theta_{n,2}, \ldots, \theta_{n,R}, \alpha_n$.

$$\mathbf{B} = [\mathbf{b}_1, \ldots, \mathbf{b}_R] \tag{2}$$
$$\hat{\mathbf{x}} = [\mathbf{B}\,\mathbf{c}_n]\boldsymbol{\theta}_n \tag{3}$$

$$p\left(\mathbf{x}_n|\mathbf{B}, \boldsymbol{\theta}_n, \mathbf{c}_n\right) = \text{Multinomial}(\mathbf{x}_n|\hat{\mathbf{x}}_n) \tag{4}$$

$$p\left(\boldsymbol{\theta}_n|\boldsymbol{\nu}\right) = \text{Dirichlet}\left(\boldsymbol{\theta}_n|\boldsymbol{\nu}\right) \tag{5}$$
$$p\left(\mathbf{c}_n|k_n, \mathbf{m}\right) = \text{Dirichlet}\left(\mathbf{c}_n|k_n\mathbf{m}\right) \tag{6}$$
$$p\left(\mathbf{m}|k', \mathbf{B}, \boldsymbol{\omega}\right) = \text{Dirichlet}\left(\mathbf{m}|k'\mathbf{B}\boldsymbol{\omega}\right) \tag{7}$$

Equation (2) represents the reference healthy profiles in matrix form, and equation (3) is simply (1) in matrix form.

In equations (5 - 7), the Dirichlet distribution is used for $\boldsymbol{\theta}_n$, $\mathbf{m}$, and $\mathbf{c}_n$, as they are all discrete probability distributions. The hyper-parameters $\boldsymbol{\nu}_n$, $k_n\mathbf{m}$, and $k'\mathbf{B}\boldsymbol{\omega}$, determine both the mean of the distribution and the strength or concentration (how peaked the distribution is near the mean).

- The easiest equation to understand is (6) where $\mathbf{m}$ is the mean of the distribution of $\mathbf{c}_n$'s, and k_n is an estimated parameter determining the strength of the distribution (and how likely it is that a particular $\mathbf{c}_n$ will be very close to the mean $\mathbf{m}$).

- Equation (7) forces the average/reference profile $\mathbf{m}$ to be close to a linear combination of normal profiles, as the cancer is likely to have a similar profile to the tissue of origin.

- For equation (5), the hyper-parameter $\boldsymbol{\nu}$ gives weights to the coefficients of the healthy and cancer profiles. The initialized coefficients give the healthy profile coefficients similar weight and a higher weight to the cancer fraction, α_n (the last entry of $\boldsymbol{\theta}_n$). However, ten additional grid searches are performed at each iteration step, adjusting these values if necessary.

The Bayesian network diagram is given in Figure 1.

The goal of the algorithm is to maximize the complete likelihood function of this model:

$$\mathbb{L} = p\left(\mathbf{m}|k', \mathbf{B}, \boldsymbol{\omega}\right) \prod_{n=1}^{N} p\left(\mathbf{c}_n|k_n, \mathbf{m}\right) p\left(\boldsymbol{\theta}_n|\boldsymbol{\nu}\right) p\left(\mathbf{x}_n|\mathbf{B}, \boldsymbol{\theta}_n, \mathbf{c}_n\right).$$

The two-step method is described in the next section.

1.2 Algorithm

The inputs to ISOpure are as follows:

- *Tumour matrix:* A matrix where the columns $\mathbf{t}_1, \mathbf{t}_2, \ldots, \mathbf{t}_N$ represent the tumor microarray profiles of N patients, where the preprocessing of the data is described in [1] and in Section **??**. In particular, the intensities should *not* be log-transformed. The size of the matrix is $G \times N$ where G is number of transcripts/features and N is the number of patients.

- *Normal matrix:* A matrix where the columns $\mathbf{b}_1, \mathbf{b}_2, \ldots, \mathbf{b}_R$ represent the microarray profiles from R normal, healthy tissue samples. The size of the matrix will be $G \times R$, where R is expected to be less than N.

The ISOpure algorithm runs in two steps, which are summarized in Figure 2. Step 1, which we will refer to as the *Cancer Profile Estimation (CPE)* step, Figure 3, estimates the average cancer profile $\mathbf{m}$, as well

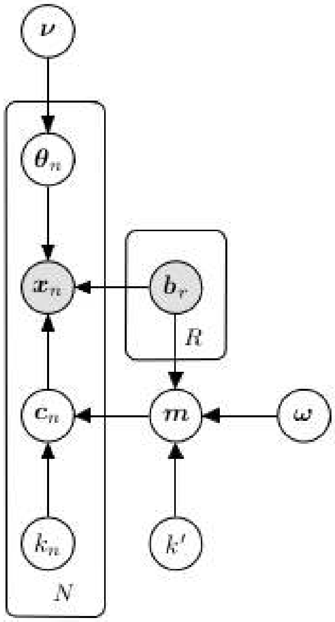

Figure 1: **Bayesian network model of ISOpure.** The graph represents the relationships between the variables in the ISOpure model, where the conditional probability of a given variable is only dependent on its parents in the graphs. The shaded circles, $\boldsymbol{x}_n$ and $\boldsymbol{b}_r$, represent the observed variables. The estimated model parameters (unshaded circles) are conditioned on the estimated model hyperparameters (also unshaded). The N and R in the corners of the plates (rectangles) indicate that the variables inside are repeated N and R times, once for each patient and healthy profile, respectively. [1]

as the cancer fraction for each patient, α_n. Since we assume that $\mathbf{c}_n = \mathbf{m}$ for all cancer profiles, the CPE step maximizes

$$\mathbb{L} = p\left(\mathbf{m}|k', \mathbf{B}, \boldsymbol{\omega}\right) \prod_{n=1}^{N} p\left(\boldsymbol{\theta}_n|\boldsymbol{\nu}\right) p\left(\mathbf{x}_n|\mathbf{B}, \boldsymbol{\theta}_n, \mathbf{m}\right).$$

Both steps perform a block coordinate descent using a conjugate gradient algorithm. The idea is to fix all variables except for one (or for a 'block' of similar variables) and minimize the negative of the log likelihood only with respect to that one (or few) variables.

In the second *Patient Profile Estimation (PPE)* step, Figure 4, the $\mathbf{c}_n$ are estimated, while $\mathbf{m}$ and α_n are kept constant to the values from the CPE step. The PPE step maximizes

$$\mathbb{L} = \prod_{n=1}^{N} p\left(\mathbf{c}_n|k_n, \mathbf{m}\right) p\left(\boldsymbol{\theta}_n|\boldsymbol{\nu}\right) p\left(\mathbf{x}_n|\mathbf{B}, \boldsymbol{\theta}_n, \mathbf{c}_n\right).$$

The outputs of *ISOpureR* at each step are lists that we have called `ISOpureS1model` and `ISOpureS2model`, respectively. They contain all the intermediate and hyper-parameters estimated by the models as well as the desired outputs. The most important entries are the following:

- The tumor 'purity' estimates, `ISOpureS1model$alphapurities` and `ISOpureS2model$alphapurities`. (The α's are estimated in the Cancer Profile Estimation step; the values of `alphapurities` from the CPE step are transferred to the model in the Patient Profile Estimation, so that `ISOpureS1model$alphapurities` and `ISOpureS2model$alphapurities` will be identical.) These are numerical vectors containing N entries, $\alpha_1, \alpha_2, \ldots, \alpha_N$, of the estimated proportion of RNA in the tumor sample that was contributed by the cancer cells, for each patient.

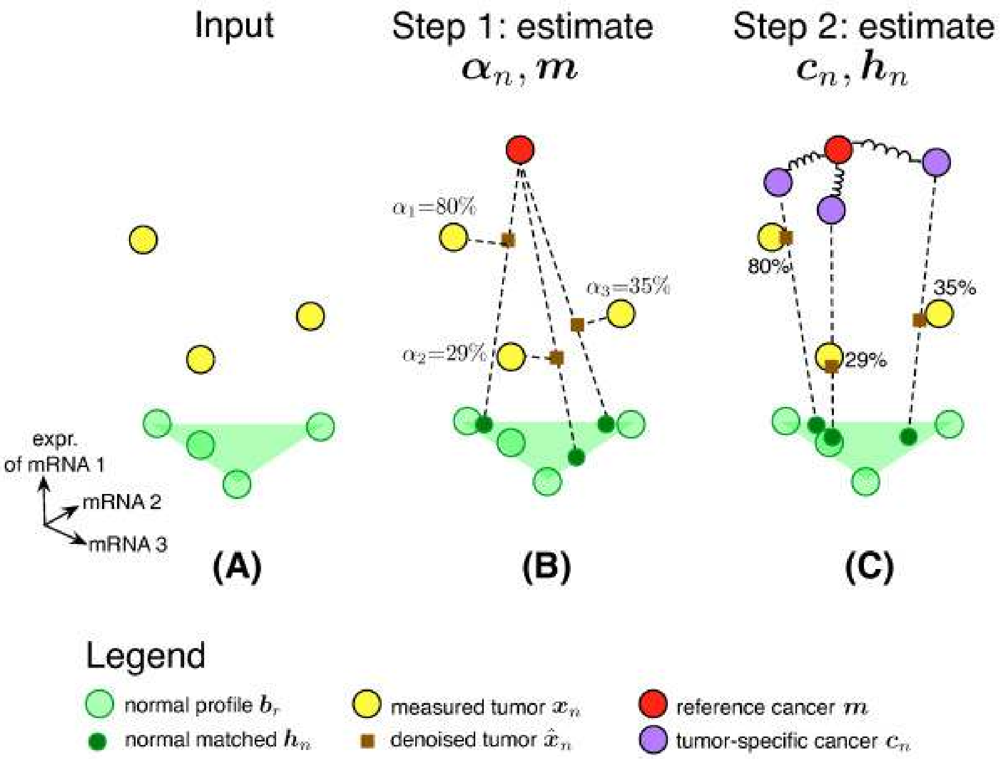

Figure 2: **ISOpure algorithm.** **(A)** The inputs to the algorithm are the tumor profiles of the patients $\{\mathbf{t}_n\}_1^N$ and healthy tissue profiles $\{\mathbf{b}_r\}_1^R$, where the $R < N$. **(B)** The *Cancer Profile Estimation (CPE)* step of ISOpure estimates the average cancer profile $\mathbf{m}$, as well as the cancer fraction for each patient, α_n. **(C)** The *Patient Profile Estimation (PPE)* step estimates the personalized cancer profile $\mathbf{c}_n$ for each patient. The values of α_n and $\mathbf{m}$ remain fixed in this step. The healthy profiles, determined by the estimation of the coefficients $\theta_{n,r}$ are estimated in the CPE step, but are re-estimated in the the PPE step. [1]

- A matrix `ISOpureS2model$cc_cancerprofiles` where the columns $\mathbf{c}_1, \mathbf{c}_2, \ldots, \mathbf{c}_N$ represent the purified cancer profiles corresponding to each mixed tumor profile. The size of the matrix will be $G \times N$, as for the tumor profiles.

2 Preprocessing steps for raw microarray data

This section will cover the processing needed to transform raw microarray data (saved as .CEL files) into the format required by *ISOpureR*. The dataset used as an example is from the Bhattacharjee et al. lung adenocarcinoma study [4], as the data is available online at `http://www.broadinstitute.org/mpr/lung/` (accessed January 20, 2015).

2.1 Load necessary libraries

The microarray assays for the Bhattacharjee dataset were performed using the Affimetrix platform. To access methods for the Affymetrix oligonucleotide arrays, the "affy" package of Bioconductor is required, and must be loaded. The details of the exact array (Affymetrix HGU95A v2) are listed in the methods of [4]; the corresponding Chip Definition File (CDF) library must also be loaded. The CDF file maps Probes (positions) into ProbeSets.

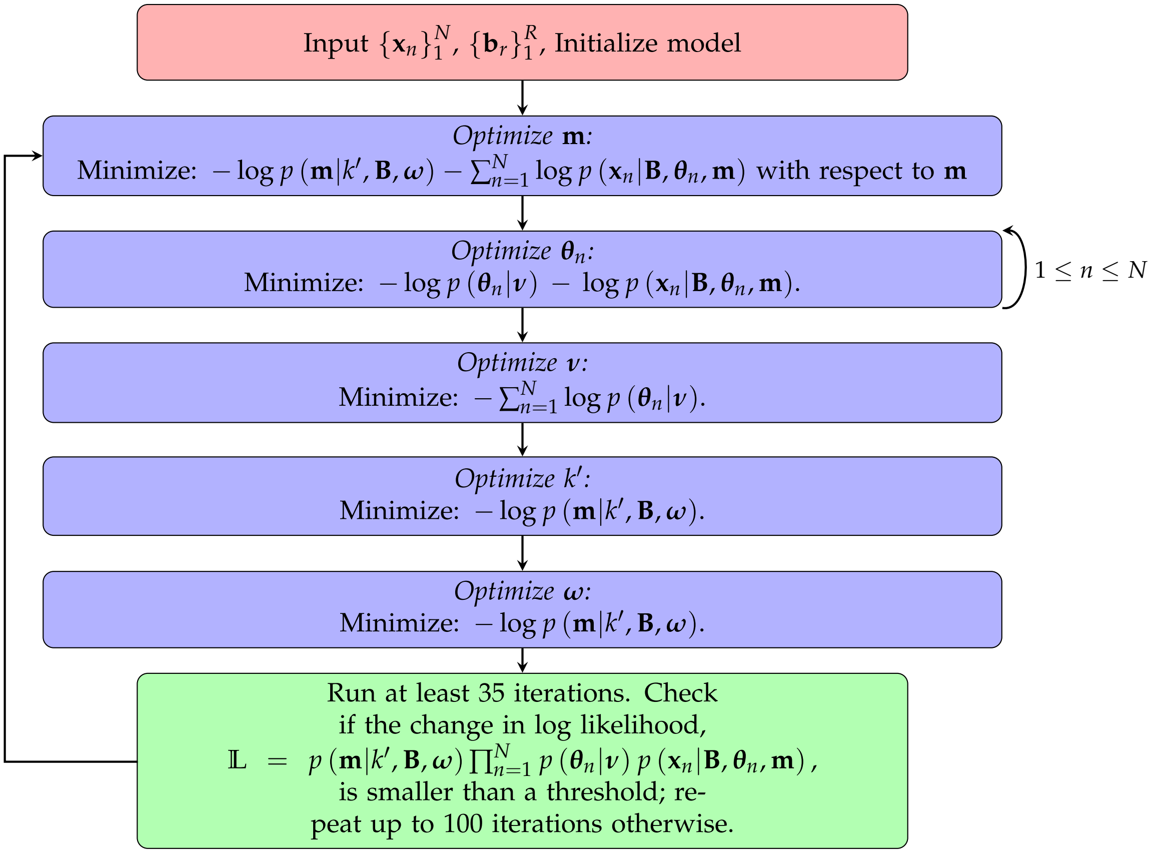

Figure 3: **Cancer Profile Estimation (CPE) step.** The flowchart illustrates the algorithm for the CPE step of ISOpure. At each step, the Polak-Ribière conjugate gradient method with Wolfe-Powell stopping criteria is used to estimate variables of the same type. Each variable is estimated once during each iteration.

```
# Install the affy library from bioconductor, if needed
# source("http://bioconductor.org/biocLite.R");
# biocLite("affy");

# Load the library
library("affy");

# Install the R CDF package for the microarray that was used for the Bhattacharjee
# dataset, the Affymetrix HG-U95A v2 platform.
# The library used is specific to the dataset analysed.
# This library also maps the probes to the Entrez Gene ID's.
library("hgu95av2hsentrezgcdf");

# Save the name of the CDF (used later)
cdf <- "hgu95av2hsentrezgcdf";
```

2.2 Process .CEL files

The tumor and normal .CEL files are located in a single directory, and will be processed together. Thus, we have downloaded the Bhattacharjee lung adenocarcinoma and normal sample .CEL files, as well as the "Array-to-sample-mapping.txt" which has information on the sample names associated to tumor/normal.

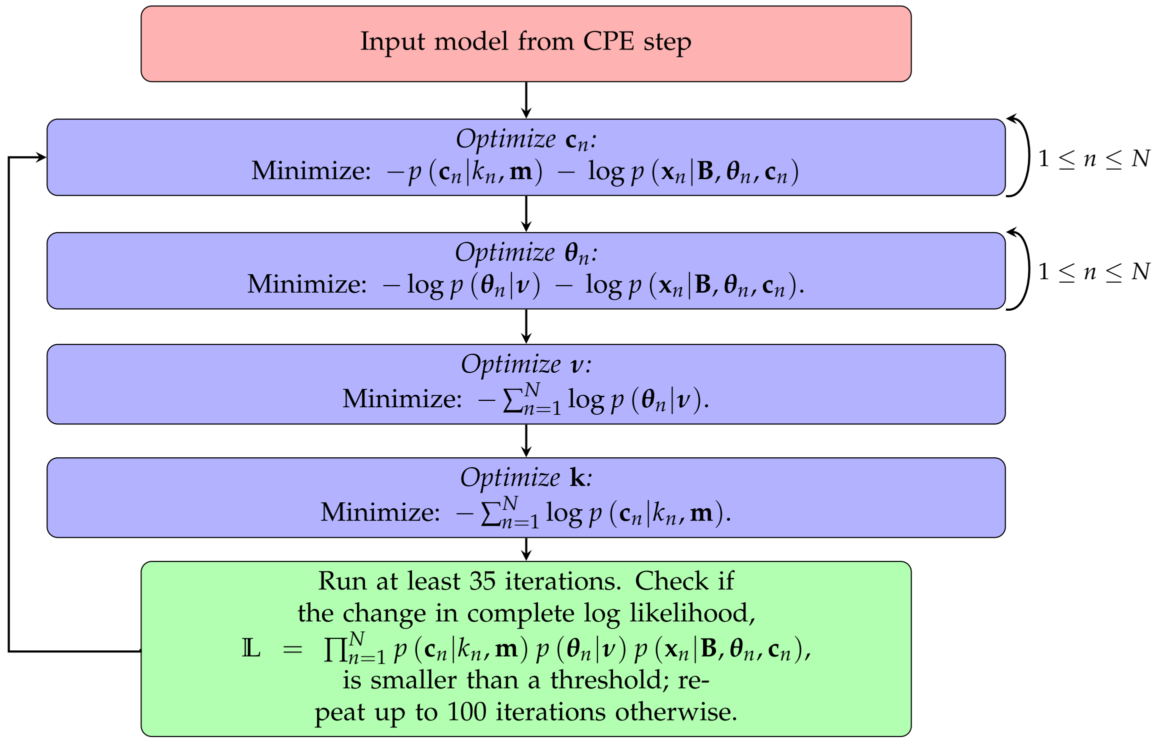

Figure 4: **Patient Profile Estimation (PPE) step.** The flowchart illustrates the algorithm for the PPE step of ISOpure. The same minimization method is used as in the CPE step. The values of α_n (the last entry of the vector $\boldsymbol{\theta}_n$) and $\mathbf{m}$ remain constant during this step.

```
# All .CEL files should be in one directory
path.to.cel.files <- file.path("~", "Datasets", "LungCancer", "Bhattacharjee",
        "raw", "CEL");

# List of all .CEL files in the directory
affy.files <- dir(
        path = path.to.cel.files,
        # match all files that begin with "CL"
        # the .CEL filenames for the Bhattacharjee dataset begin with this string
        pattern = "^CL",
        # return only names of visible files
        all.files=FALSE,
        # return only file names, not relative file paths
        full.names=FALSE,
        # assume all are in given directory, not in any subdirectories
        recursive=FALSE,
        ignore.case=TRUE
        )
```

Now we can return the expression values for those files. However, the `exprs` function returns $\log_2$ mRNA abundance values. For ISOpure, we need the normalized count of the number of copies of each transcript present in the sample, so the values are exponentiated.

```
# Change to directory where affy files are located, as it will be easier to run
# justRMA thete
setwd(path.to.cel.files);
```

```r
# Read .CEL files into an expression set
rma.results <- justRMA(
        filenames = affy.files,
        cdfname = cdf
        );

# Return to working directory
setwd(base.dir);

# Return just the expression values
# This will give you the log2 mRNA abundance dataset
all.expression.data.log <- exprs(rma.results);

# non-log-transformed data
all.expression.data <- 2^(all.expression.data.log);
```

2.3 Obtain tumor and normal expression data

Now, we just need to separate the data into the tumour and normal expression profiles. First use the array-to-sample mapping file to select the normal sample names.

```r
path.to.sample.info <- file.path("~", "Datasets", "LungCancer", "Bhattacharjee",
        "raw", "Array-to-sample-mapping.txt");

# read in array-to-sample info into a dataframe
sample.info <- read.table(
        file = path.to.sample.info,
        sep = "\t",
        header = TRUE,
        as.is = TRUE
        );

# find which sample names correspond to normal samples and tumor samples
normal.sample.names <- sample.info$scan[which("NORMAL"==sample.info$CLASS)];
```

Finally, separate the expression data.

```r
# select only the columns from all the expression data with filenames for
# the normal samples -- had to concatenate .CEL to the end
normal.expression.data <- all.expression.data[ , paste0(normal.sample.names, '.CEL')];

# omit all the columns with sample names from normal samples, to obtain
# all tumour samples
tumor.expression.data <- all.expression.data[,
        !(colnames(all.expression.data) %in% paste0(normal.sample.names, '.CEL'))];
```

2.4 Further processing

The Bhattacharjee dataset has several replicates per patient (see the key to scan names in the online data) and for the data used in [1] and [3], these were averaged. There may be further processing required for a given dataset; more information on the Affymetrix oligonucleotide arrays can be found in the Bioconductor `affy` package [5]. To simplify the example, we will apply *ISOpureR* to all the replicates after the computation the RMA expression values and exponentiation performed in the code above, in Section 2.2.

2.5 Save expression data

To save the expression data to file, use the `write.table` function in R, as below.

```
path.to.expression.dir <- file.path("~", "Datasets", "LungCancer", "Bhattacharjee",
        "expression");

print("Writing tumor expression matrix...");
write.table(
        x = tumor.expression.data,
        file = file.path(path.to.expression.dir, "Bhattacharjee_tumor_mRNA_abundance.txt"),
        sep = "\t"
        );

print("Writing tumor expression matrix...");
write.table(
        x = normal.expression.data,
        file = file.path(path.to.expression.dir, "Bhattacharjee_normal_mRNA_abundance.txt"),
        sep = "\t"
        );
```

2.6 Run *ISOpureR* on the expression data

The following section will outline the functions that run the *ISOpureR* model on the data processed above. This example takes a long time to run. Section 3 describes a smaller example applied to data included in the *ISOpureR* library, with a shorter running time.

If we have just performed the microarray preprocessing, we can begin by loading the library and checking that both the tumor and normal expression data is in matrix form before running *ISOpureR*.

```
# Load ISOpureR library
library(ISOpureR);

# Check that data is in matrix form
is.matrix(tumor.expression.data);
# [1] TRUE
is.matrix(normal.expression.data);
# [1] TRUE
```

To run *ISOpureR* from saved data, the only additional steps are to load the data from the file created in Section 2.5, which will result in two dataframes, and change these to matrices.

```
# Load ISOpureR library
library(ISOpureR);

# Define path to the expression data
path.to.expression.dir <- file.path("~", "Datasets", "LungCancer", "Bhattacharjee",
        "expression");

# Load normal and tumor expression data
normal.expression.data <- read.table(
        file.path(path.to.expression.dir, "Bhattacharjee_normal_mRNA_abundance.txt"),
        header=TRUE,
        sep = "\t"
        );
```

```
tumor.expression.data <- read.table(
        file.path(path.to.expression.dir, "Bhattacharjee_tumor_mRNA_abundance.txt"),
        header=TRUE,
        sep = "\t"
        );

# Make sure that data is in matrix form, since
# read.table() will load the data as dataframe
normal.expression.data <- as.matrix(normal.expression.data);
tumor.expression.data <- as.matrix(tumor.expression.data);
```

Now, in either case, we can run the two functions for the Cancer Profile Estimation and the Patient Profile Estimation of *ISOpureR*. More details about these functions are given in Section 3. Aside from checking that the expression levels are all greater than 1, you may also want to check that the sum of the elements is similar across all profiles. Finally, the tumour profiles should also be discretized according to the statistical model, but the likelihood calculation should work without this step.

```
# Check that expression levels are all greater than 1
# As well, the minimum should not return NA or NaN
min(normal.expression.data);
min(tumor.expression.data);

# For reproducible results, set the random seed
set.seed(123);
# Run ISOpureR Step 1 - Cancer Profile Estimation
ISOpureS1model <- ISOpure.step1.CPE(
        tumor.expression.data,
        normal.expression.data
        );

# For reproducible results, set the random seed
set.seed(456);
# Run ISOpureR Step 2 - Patient Profile Estimation
ISOpureS2model <- ISOpure.step2.PPE(
        tumor.expression.data,
        normal.expression.data,
        ISOpureS1model
        );
```

3 Applying *ISOpureR* to a small example

We will show how to use *ISOpureR* with a small part of the lung adenocarcinoma expression data from Beer et al. [6], which is included with the package. Although the full dataset contains measured expression levels of 5151 transcripts in 86 patients, only data from 1000 transcripts and 30 patients is included with *ISOpureR* to reduce the file size. The dataset also contains 10 reference healthy samples.

3.1 Load and format data

The first step is to load the data and make sure that both the tumor data and the normal data are in matrix form, with the required dimensions.

```
# Load the library and the data included with the package
# Data is not the full Beer dataset due to memory constraints
library(ISOpureR);
path.to.data <- paste0(file.path(system.file(package = "ISOpureR"), 'extdata', 'Beer'));
load(file.path(path.to.data , 'beer.normaldata.1000.transcripts.RData'));
load(file.path(path.to.data , 'beer.tumordata.1000.transcripts.30.patients.RData'));

# Check what the data looks like
# The tumor data is rather large, so just look at the normal data for this example
str(beer.normaldata);

##  num [1:1000, 1:10] 231 1200 159 850 195 ...
##  - attr(*, "dimnames")=List of 2
##   ..$ : chr [1:1000] "10_at" "100_at" "1000_at" "10007_at" ...
##   ..$ : chr [1:10] "N1" "N2" "N3" "N4" ...

# Make sure that everything is in matrix form
beer.normaldata <- as.matrix(beer.normaldata);
beer.tumordata <- as.matrix(beer.tumordata);

# Check that the minimum values are greater than 1, not NA, NaN
min(beer.normaldata);

## [1] 123.0638

min(beer.tumordata);

## [1] 114.923

# You may also want to check that the sums of the entries are similar
apply(beer.normaldata, 2, sum);

##      N1      N2      N3      N4      N5      N6      N7      N8      N9     N10
## 1137194 1169377 1139336 1179519 1176447 1148149 1250439 1139013 1126210 1147247

apply(beer.tumordata, 2, sum);

##         1         2         3         4         5         6         7         8         9
## 1076851.2 1100111.6 1211734.9 1202102.7 1122013.1 1201160.3 1048613.9 1151243.8 1170046.0
##        10        11        12        13        14        15        16        17        18
## 1127474.9 1186974.6 1130150.3 1092075.1 1202368.1 1033596.4 1078185.8  900539.3 1153557.3
##        19        20        21        22        23        24        25        26        27
## 1097983.4 1145483.4 1193260.9 1084389.6 1108325.0 1143825.7 1120305.2 1013043.9 1189709.6
##        28        29        30
## 1188708.4 1178579.0 1153720.5

# Check what the data looks like
str(beer.normaldata);

##  num [1:1000, 1:10] 231 1200 159 850 195 ...
##  - attr(*, "dimnames")=List of 2
##   ..$ : chr [1:1000] "10_at" "100_at" "1000_at" "10007_at" ...
##   ..$ : chr [1:10] "N1" "N2" "N3" "N4" ...

str(beer.tumordata);

##  num [1:1000, 1:30] 218 1399 155 893 170 ...
##  - attr(*, "dimnames")=List of 2
##   ..$ : chr [1:1000] "10_at" "100_at" "1000_at" "10007_at" ...
##   ..$ : chr [1:30] "1" "2" "3" "4" ...
```

3.2 Run the Cancer Profile Estimation

The ISOpure model runs in two steps. To perform the first step of ISOpure, which will estimate the proportion of cancer cells, α_n, for each patient, as well as an average cancer profile, type the following:

```
# For reproducible results, set the random seed
set.seed(123);
# Run ISOpureR Step 1  - Cancer Profile Estimation
ISOpureS1model <- ISOpure.step1.CPE(beer.tumordata, beer.normaldata);
```

Note that the function names of these steps have changed from the MATLAB implementation, and from earlier versions of *ISOpureR* (before v.1.0.14). The run time for both steps is about 20-30 minutes for the small dataset (but can take several days for large datasets). The output to the screen will look something like this:

```
# [1] "---------------------"
# [1] "Initializing..."
# [1] "MIN_KAPPA set to  9571.36859512808"
# [1] "--- optimizing mm..."
# [1] "--- optimizing theta..."
# [1] "--- optimizing vv..."
# [1] "--- optimizing kappa..."
# [1] "--- optimizing omega..."
# [1] "Total log likelihood: -213825284.681581"
# [1] "iter:  1 / 35 , loglikelihood:  -213825284.681581 , change:  Inf"
# [1] "--- optimizing mm..."
# [1] "--- optimizing theta..."
# [1] "--- optimizing vv..."
# [1] "--- optimizing kappa..."
# [1] "--- optimizing omega..."
# [1] "Total log likelihood: -213802936.615107"
# [1] "iter:  2 / 35 , loglikelihood:  -213802936.615107 , change:  0.000104526471096462"
# [1] "--- optimizing mm..."
# [1] "--- optimizing theta..."
# [1] "--- optimizing vv..."
# [1] "--- optimizing kappa..."
# [1] "--- optimizing omega..."
# [1] "Total log likelihood: -213796857.989424"
# [1] "iter:  3 / 35 , loglikelihood:  -213796857.989424 , change:  2.84317821142625e-05"
```

The optimization of the loglikelihood will run for at least 35 iterations, and if the change in loglikelihood is greater than 10^{-7}, up to 100 iterations. At the end of this process you may see warnings as below.

```
warnings()
# Warning messages:
# 1: In sqrt(B * B - A * d1 * (x2 - x1)) : NaNs produced
# 2: In sqrt(B * B - A * d1 * (x2 - x1)) : NaNs produced
# 3: In sqrt(B * B - A * d1 * (x2 - x1)) : NaNs produced
```

These are nothing to worry about. They are part of the optimization calculations, and when a `NaN` is produced, the algorithm detects that it has not converged and simply adjusts the step size.

The list `ISOpureS1model` which is returned will contain all the information on parameters estimated from the first step. If you would like to see what the list looks like without performing all the calculations, you can load the saved result of the Cancer Profile Estimation from the data folder. The most important list entry is the vector of estimated fractions of cancer content, `alphapurities`.

```r
# Load the saved ISOpureS1model for this example, if time is an issue
load(file.path(path.to.data , 'beer.ISOpureS1model.1000.transcripts.30.patients.RData'));
ls();
```

```
## [1] "ISOpureS1model"  "beer.normaldata" "beer.tumordata"  "path.to.data"
```

```r
# Check that what ISOpureS1model looks like
str(ISOpureS1model);
```

```
## List of 14
##  $ vv                : num [1, 1:11] 1 1 1 1 1 ...
##  $ log_BBtranspose    : num [1:10, 1:1000] -8.5 -8.57 -8.58 -8.58 -8.62 ...
##   ..- attr(*, "dimnames")=List of 2
##   .. ..$ : chr [1:10] "N1" "N2" "N3" "N4" ...
##   .. ..$ : chr [1:1000] "10_at" "100_at" "1000_at" "10007_at" ...
##  $ PPtranspose        : num [1:10, 1:1000] 0.000203 0.00019 0.000188 0.000187 0.000181 ...
##   ..- attr(*, "dimnames")=List of 2
##   .. ..$ : chr [1:10] "N1" "N2" "N3" "N4" ...
##   .. ..$ : chr [1:1000] "10_at" "100_at" "1000_at" "10007_at" ...
##  $ kappa              : num 9571
##  $ theta              : num [1:30, 1:11] 1.47e-06 6.34e-07 2.94e-09 1.82e-10 2.47e-02 ...
##  $ omega              : num [1:10, 1] 6.51e-01 2.41e-09 4.09e-27 3.49e-01 6.41e-28 ...
##  $ log_all_rates      : num [1:11, 1:1000] -8.5 -8.57 -8.58 -8.58 -8.62 ...
##   ..- attr(*, "dimnames")=List of 2
##   .. ..$ : chr [1:11] "N1" "N2" "N3" "N4" ...
##   .. ..$ : chr [1:1000] "10_at" "100_at" "1000_at" "10007_at" ...
##  $ MIN_KAPPA          : num 9571
##  $ total_loglikelihood: num [1, 1] -2.14e+08
##   ..- attr(*, "dimnames")=List of 2
##   .. ..$ : NULL
##   .. ..$ : chr ""
##  $ mm_weights         : num [1, 1:1000] -8.57 -6.72 -8.88 -6.97 -8.64 ...
##  $ theta_weights      : num [1:30, 1:11] -3 -3 -3 -3 -3 ...
##  $ omega_weights      : num [1:10, 1] -2.3 -21.72 -62.63 -2.92 -64.49 ...
##  $ mm                 : Named num [1:1000] 0.000195 0.001237 0.000143 0.000967 0.000182 ...
##   ..- attr(*, "names")= chr [1:1000] "10_at" "100_at" "1000_at" "10007_at" ...
##  $ alphapurities      : num [1:30] 0.682 0.735 0.662 0.481 0.701 ...
```

```r
# Look at the alphapurities vector in particular
ISOpureS1model$alphapurities;
```

```
##  [1] 0.6823474 0.7347548 0.6618625 0.4808950 0.7007916 0.4433997 0.6725943 0.7770067
##  [9] 0.5273706 0.8552701 0.6604500 0.8124706 0.6201061 0.6084832 0.7198680 0.4430942
## [17] 0.8495830 0.5502081 0.5742360 0.5408939 0.2973461 0.6542631 0.7352760 0.7359806
## [25] 0.5905198 0.7723543 0.5165743 0.7074744 0.6105623 0.5933992
```

3.3 Run the Patient Profile Estimation

Once the Cancer Profile Estimation is complete, to perform the second step of ISOpure, which will estimate the patient-specific cancer mRNA expression profiles, call the following function:

```r
# For reproducible results, set the random seed
set.seed(456);
```

```r
# Run ISOpureR Step 2 - Patient Profile Estimation
ISOpureS2model <- ISOpure.step2.PPE(
                                        beer.tumordata,
                                        beer.normaldata,
                                        ISOpureS1model
                                        );
```

The screen output will look very similar to the output in the previous step.

```
# [1] "---------------------"
# [1] "Initializing..."
# [1] "MIN_KAPPA set to  669612.15072434"
# [1] "--- optimizing cc..."
# [1] "--- optimizing theta..."
# [1] "--- optimizing vv..."
# [1] "--- optimizing kappa..."
# [1] "Total log likelihood: -213314739.637299"
# [1] "iter:  1 / 35 , loglikelihood:  -213314739.637299 , change:  Inf"
# [1] "--- optimizing cc..."
# [1] "--- optimizing theta..."
# [1] "--- optimizing vv..."
# [1] "--- optimizing kappa..."
# [1] "Total log likelihood: -212880711.000007"
# [1] "iter:  2 / 35 , loglikelihood:  -212880711.000007 , change:  0.00203883496655811"
```

Again, `ISOpureS2model` which is returned by the function `ISOpure.step2.PPE` will contain all the information on parameters estimated in the Patient Profile Estimation step. The matrix `ISOpureS2model$cc_cancerprofiles` will contain the patient-specific cancer profiles and is of the same dimension as the `tumordata` matrix. It is also of the same scale, (*i.e.* although ISOpureS2 treats purified cancer profiles as parameters of a multinomial distribution, we re-scale them to be on the same scale as the input tumor profiles). The n-th column corresponds to the profile for the n-th patient.

```r
# Load the saved ISOpureS2model for this example, if time is an issue
# Note that the data has been rounded to 5 decimals (for CRAN memory requirements)
load(file.path(
                path.to.data,
                'beer.ISOpureS2model.1000.transcripts.30.patients.rounded.RData'
                )
        );

# Check that what ISOpureS2model looks like
str(ISOpureS2model);

## List of 13
##  $ vv                   : num [1, 1:11] 1 1 1 1 1 1 1 1 1 1 ...
##  $ log_BBtranspose      : num [1:10, 1:1000] -8.5 -8.57 -8.58 -8.58 -8.62 ...
##   ..- attr(*, "dimnames")=List of 2
##   .. ..$ : chr [1:10] "N1" "N2" "N3" "N4" ...
##   .. ..$ : chr [1:1000] "10_at" "100_at" "1000_at" "10007_at" ...
##  $ PPtranspose          : num [1, 1:1000] 0.000195 0.001237 0.000143 0.000967 0.000182 ...
##  $ kappa                : num [1, 1:30] 669612 669612 669612 669612 669612 ...
##  $ omega                : num [1:30, 1] 1 1 1 1 1 1 1 1 1 1 ...
##  $ log_cc               : num [1:30, 1:1000] -8.35 -8.18 -8.46 -8.19 -8.19 ...
##  $ MIN_KAPPA            : num 669612
```

```
##  $ total_loglikelihood: num [1, 1] -2.13e+08
##  $ theta              : num [1:30, 1:11] 2.37e-07 1.10e-06 2.88e-08 1.14e-18 3.95e-02 ...
##  $ cc_weights         : num [1:30, 1:1000] 0.000195 0.000195 0.000195 0.000195 0.000195 ...
##  $ theta_weights      : num [1:30, 1:10] -13.4 -14.3 -19.6 -22.4 -3.7 ...
##  $ alphapurities      : num [1:30] 0.682 0.735 0.662 0.481 0.701 ...
##  $ cc_cancerprofiles  : num [1:1000, 1:30] 254 1408 155 956 185 ...

# Check what the cancer profiles look like
str(ISOpureS2model$cc_cancerprofiles);

##  num [1:1000, 1:30] 254 1408 155 956 185 ...

# Look at the first entries in the cancer profile for a particular patient,
# say patient 3
head(ISOpureS2model$cc_cancerprofiles[ ,3]);

## [1]  257.20 1511.80  157.74 1045.60  191.02 1353.50
```

4 Visualize results from small example

This section gives simple examples for visualizing the output from *ISOpureR*, using base R plotting functions. The dataset included in the package is incomplete; thus the figures are for illustrative purposes only, and the choice of plotting functions will be directed by the analysis required for a specific project. In particular, the main use of *ISOpureR* has been to generate purified profiles which can be used for prognostic biomarkers, summarized in Section 5. Before starting, we must load the data.

```
# Load data
library(ISOpureR);

path.to.data <- paste0(file.path(system.file(package = "ISOpureR"), 'extdata', 'Beer'));
load(file.path(path.to.data , 'beer.tumordata.1000.transcripts.30.patients.RData'));
load(file.path(path.to.data , 'beer.normaldata.1000.transcripts.RData'));
load(file.path(path.to.data , 'beer.ISOpureS2model.1000.transcripts.30.patients.rounded.RData'));
```

4.1 Proportion of cancer

The first graph is a histogram of the cancer proportions in the patients' tumors, estimated using ISOpure.

```
beer.cellularity <- ISOpureS2model$alphapurities;

# Histogram of the estimated proportions of cancer in the samples
hist(
        x = beer.cellularity,
        main = "Tumour cellularity histogram",
        xlab = "Proportion of cancer",
        );
```

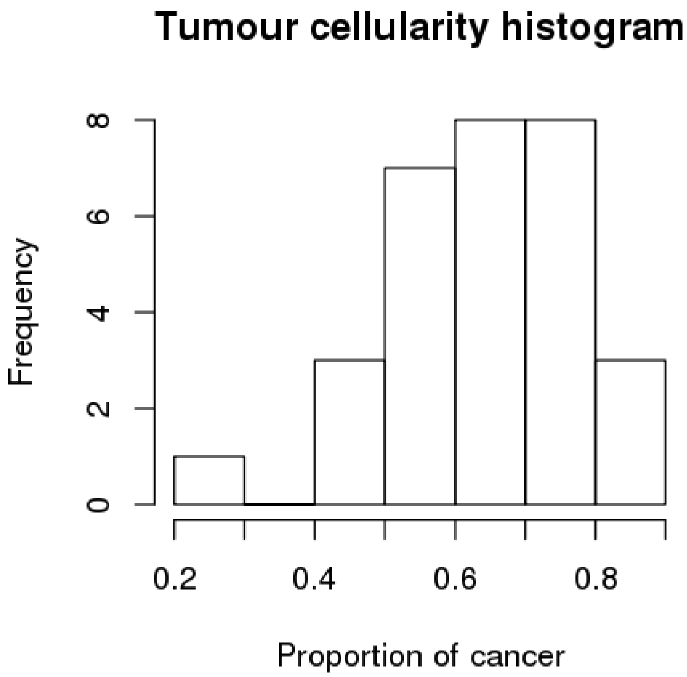

If estimates of the tumor cellularity are available from pathologists, it may be useful to compare the estimates from the pathologists with the estimated proportions from ISOpureR. Since for the Beer dataset we do not have pathologist estimates of cancer fraction, we compare instead the fractions estimated by the algorithm using the full Beer dataset to the smaller dataset included in the *ISOpureR* package.

```
# Load estimated proportions from full dataset
alphapurities.full.dataset <- read.table(
        file=file.path(path.to.data, "alphapurities_full_dataset.txt"),
        sep="\t",
        header = TRUE
        );

# Plot comparison of proportions estimated with package dataset versus
# 'real' proportions estimated with full Beer dataset, both using ISOpureR.
# If pathologist estimates exist for the dataset, use those as the 'real' values.

# Make title smaller
par(cex.main = 1);
plot(
        x = alphapurities.full.dataset$alphapurities,
        y = ISOpureS2model$alphapurities,
        asp = 1,
        xlab = expression(paste(alpha, ", full Beer dataset")),
        ylab = expression(paste(alpha, ", ISOpureR package dataset")),
        main = "Comparison of estimates with ISOpureR"
        );
```

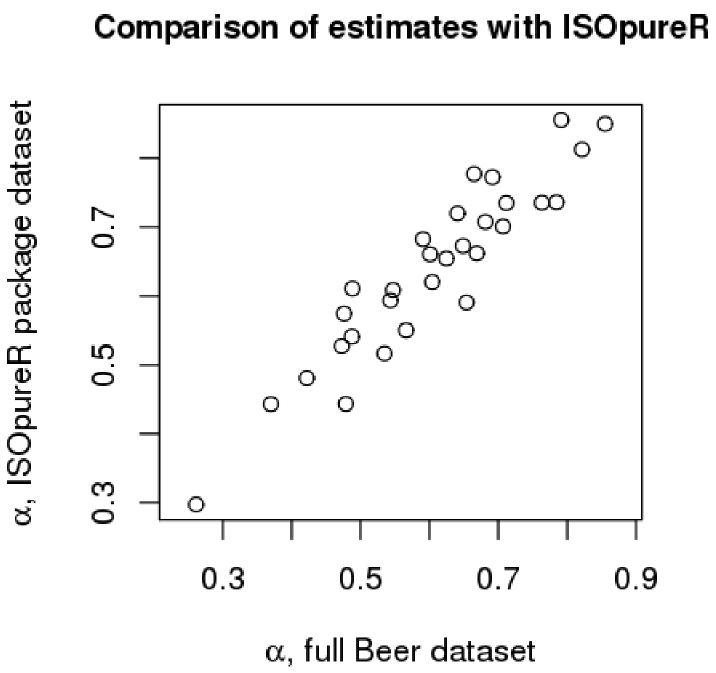

4.2 Tumor and cancer profiles

The following two figures are of heatmaps of the tumor profiles (*i.e.* pre-purification) and of the cancer profiles (*i.e.* post-purification). Even with 1000 probes, the heatmaps are cluttered, thus the log intensity for only the first 100 probes is shown for each.

```
# Define nicer heatmap colours
heatmapcol <- topo.colors(100);

# Choose just the first 100 probes, as there are too many to plot all
beer.tumordata.subset <- beer.tumordata[1:100, ];

# Plot heatmap of tumor profiles
heatmap(
        x = log2(beer.tumordata.subset),
        margins = c(3,5),
        col = heatmapcol,
        xlab = "Patient ID",
        ylab = "Probes",
        cexRow = 0.5
        );
```

The code for generating the heatmap for the cancer profiles is very similar.

```
# Look at just the cancer profiles from the data produced by the Patient Profile
# Estimation step
beer.cancerdata <- ISOpureS2model$cc_cancerprofiles;
colnames(beer.cancerdata) <- 1:30;
```

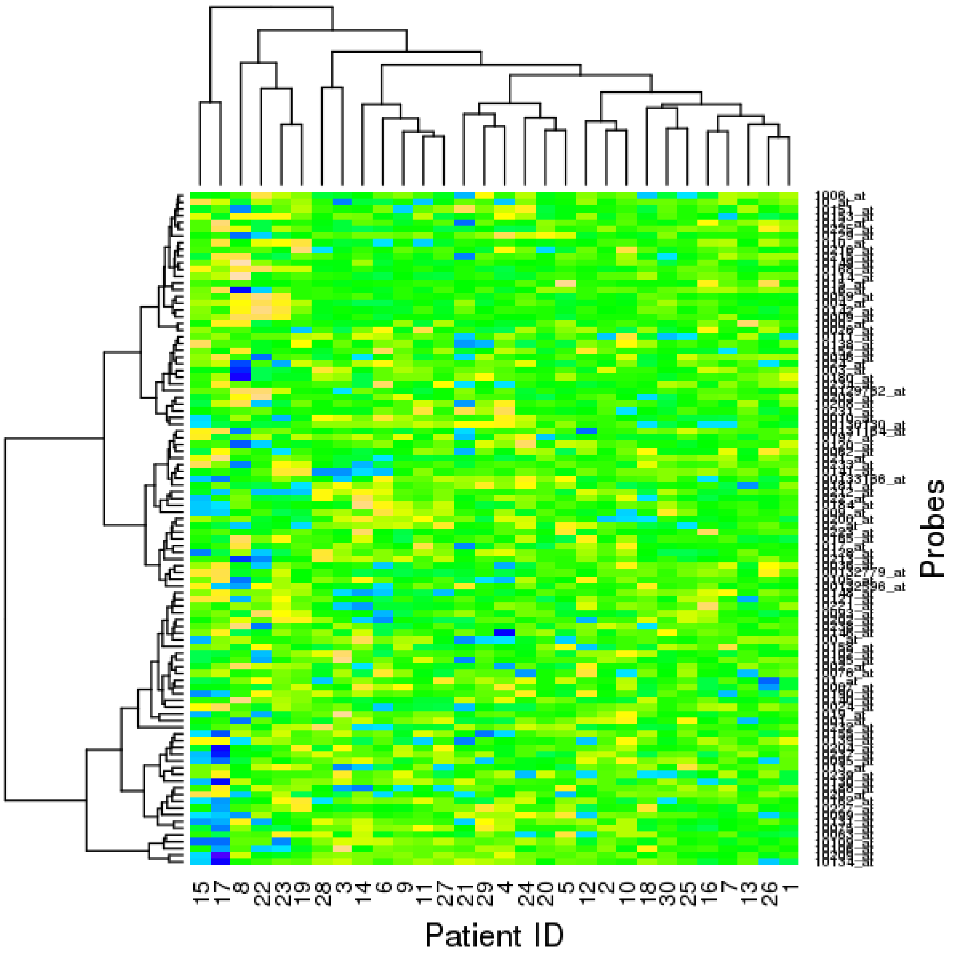

Figure 5: Tumor mRNA abundance profiles. Only 100 probes are shown, the log intensity is used as input, and the data is automatically scaled by the heatmap function.

```
probeset.names <- read.table(
        file.path(path.to.data, "probeset_names.txt"),
        header = FALSE,
        col.names=c("probe"),
        as.is = TRUE
        );
rownames(beer.cancerdata) <- probeset.names$probe;

# Choose just the first 100 probes, as there are too many to plot all
beer.cancerdata.subset <- beer.cancerdata[1:100, ];

# Plot heatmap of cancer profiles
heatmap(
        x = log2(beer.cancerdata.subset),
        margins = c(3,5),
        col = heatmapcol,
        xlab = "Patient ID",
        ylab = "Probes",
        cexRow = 0.5
        );
```

The previous heatmaps are not very useful for interpreting the data; including all probesets prevents the probeset names from being distinguishable on the graph, and it is difficult to quantify the differences between the tumor and cancer heatmaps.

One possible example of an analysis is to look at the genes differentially expressed between the tumor and the normal profiles, and again at those differentially expressed between the ISOpure-purified cancer profiles and the normal profiles. The following code runs though this analysis, using the limma package [7] and inspired from [8]. However, recall that this is simply a toy example, as we are using the small dataset included in the package.

```
# Install the limma package from bioconductor, if needed
source("http://bioconductor.org/biocLite.R");
biocLite("limma");

# Load the library
library("limma");

# Concatenate tumor and normal data into a large expression matrix
expr.data <- log2(cbind(beer.tumordata, beer.normaldata));

# First 30 profiles correspond to tumour, last 10 to normal
pheno.data <- data.frame(
        Tumor = factor(c(rep("Yes", 30), rep("No", 10)))
        );

# Create design matrix
design   <- model.matrix(~ -1 + Tumor, pheno.data);
contrast <- makeContrasts(TumorYes-TumorNo, levels = design);

# Fit the linear model
fit1 <-lmFit(expr.data, design);
fit2 <- contrasts.fit(fit1, contrast);
fit3 <- eBayes(fit2);
```

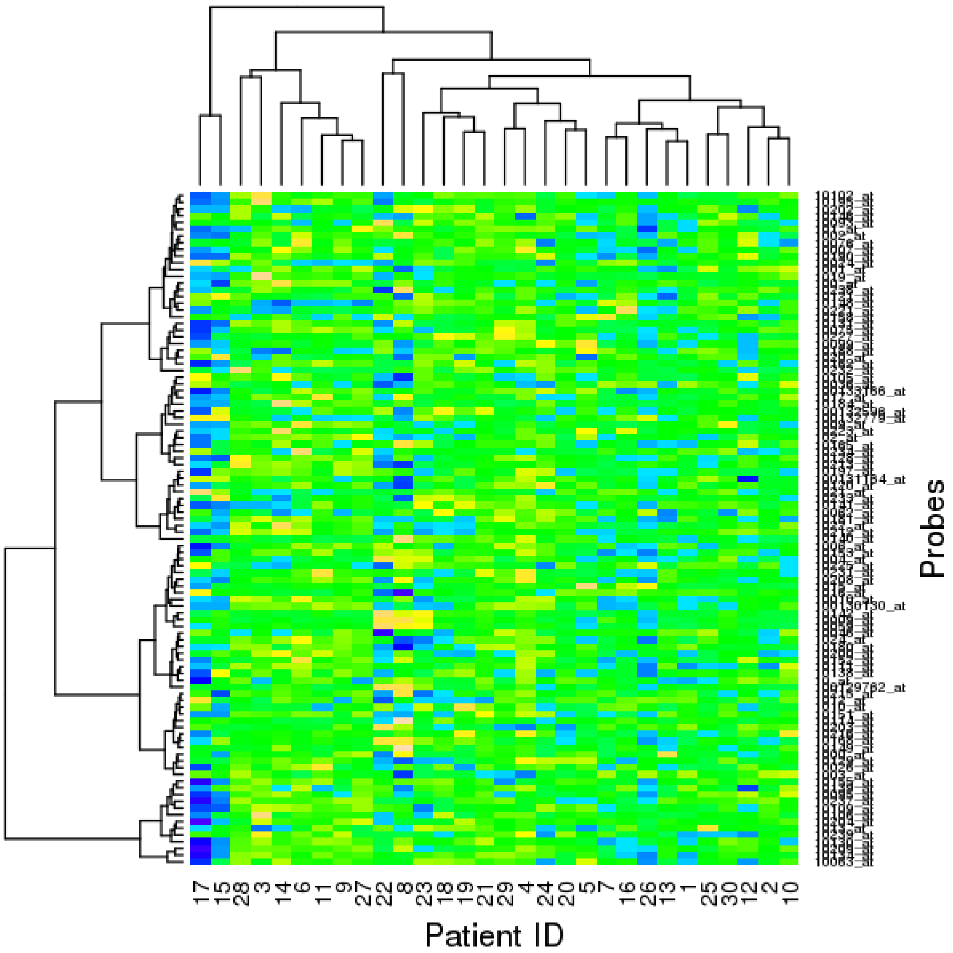

Figure 6: Cancer mRNA abundance profiles. Only 100 probes are shown, the log intensity is used as input, and the data is automatically scaled by the heatmap function.

```
# Select only genes that are differentially expressed between tumor
# and normal, default using the Holm method
selected  <- p.adjust(fit3$p.value) < 0.05;
expr.selected <- expr.data[selected, ];

# Add an color bar indicating whether the sample is tumor or normal
color.map <- function(tumor.status) { if (tumor.status=="Yes") "#FF0000" else "#0000FF" };
patientcolors <- unlist(lapply(pheno.data$Tumor, color.map));

# Draw heatmap
heatmap(
        x = expr.selected,
        margins = c(3,4.5),
        col = heatmapcol,
        xlab = "Patient ID",
        ylab = "Probes",
        cexRow = 0.5,
        ColSideColors=patientcolors
        );
```

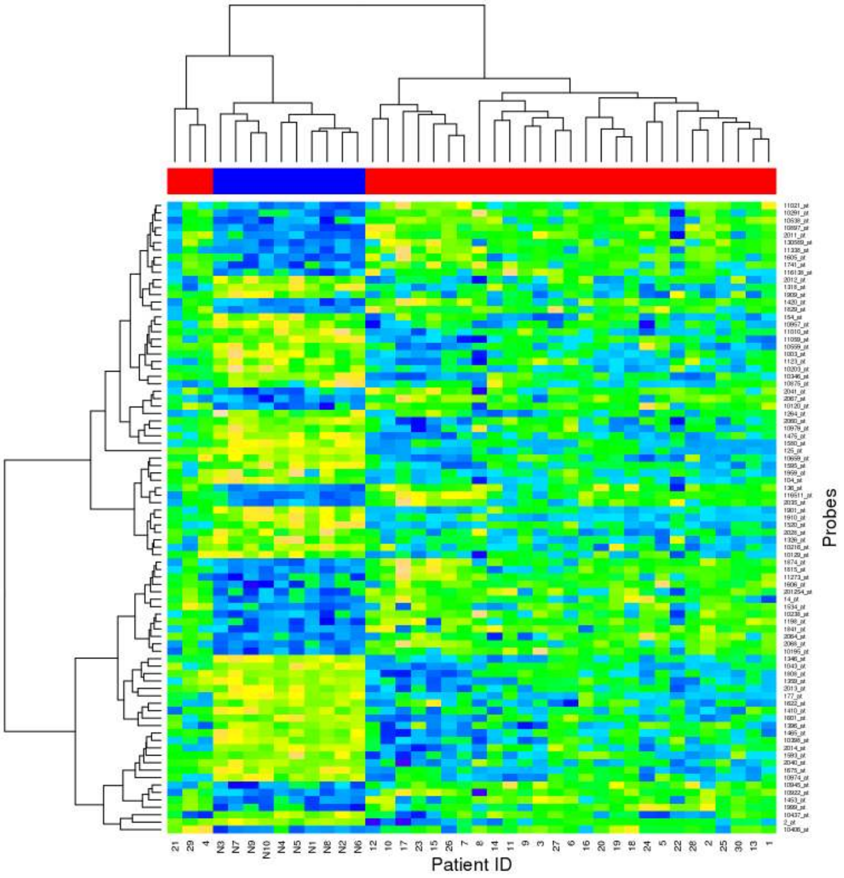

Figure 7: A heatmap comparing differential expression between tumour samples (red columns) and normal samples (blue columns). Only 85 transcript levels are differentially expressed between cancer and normal, Holm-adjusted p-value of 0.05.

The code for comparing differential expression between purified cancer profiles and normal profiles is

similar.

```
# Concatenate cancer and normal data into a large expression matrix
expr.data <- log2(cbind(beer.cancerdata, beer.normaldata));
pheno.data <- data.frame(
        Tumor = factor(c(rep("Yes", 30), rep("No", 10)))
        );

# Fit the linear model
design   <- model.matrix(~ -1 + Tumor, pheno.data);
contrast <- makeContrasts(TumorYes-TumorNo, levels = design);

fit1 <-lmFit(expr.data, design);
fit2 <- contrasts.fit(fit1, contrast);
fit3 <- eBayes(fit2);

# More genes are differentially expressed, so decrease the threshold
selected  <- p.adjust(fit3$p.value) < 0.01;
expr.selected <- expr.data[selected, ];

# Draw heatmap
heatmap(
        x = expr.selected,
        margins = c(3,2),
        col = heatmapcol,
        xlab = "Patient ID",
        ylab = "Probes",
        # too many genes to show
        labRow = "",
        ColSideColors=patientcolors
        );
```

5 Generating gene signatures

Purified cancer profiles have been shown to generate better prognostic gene signatures compared to mixed tumor profiles [1]. The purified cancer profiles $\mathbf{c}_n$ (rather than the mixed tumor profiles $\mathbf{t}_n$) were used to train a Cox proportional hazards (CPH) model to predict survival data for each patient. To test, another dataset of samples were purified using ISOpure and then used to compute the risk for each patient, using the CPH model parameters learned on the training set.

References

[1] Quon, G., Haider, S., Deshwar, A.G., Cui, A., Boutros, P.C., Morris, Q., Computational purification of individual tumor gene expression profiles leads to significant improvements in prognostic prediction. Genome Medicine, 5:29 (2013). http://www.ncbi.nlm.nih.gov/pubmed/23537167.

[2] Quon, G., Morris, Q., ISOLATE: A computational strategy for identifying the primary origin of cancers using high-throughput sequencing. Bioinformatics, 25:2882-2889 (2009) http://www.ncbi.nlm.nih.gov/pubmed/19542156

[3] (submitted) Anghel C.V., Quon, G. Haider S., Nguyen F., Deshwar A.G., Morris Q.D., Boutros P.C., ISOpureR: an R implementation of a computational purification algorithm of mixed tumor profiles. BMC Bioinformatics. (2015)

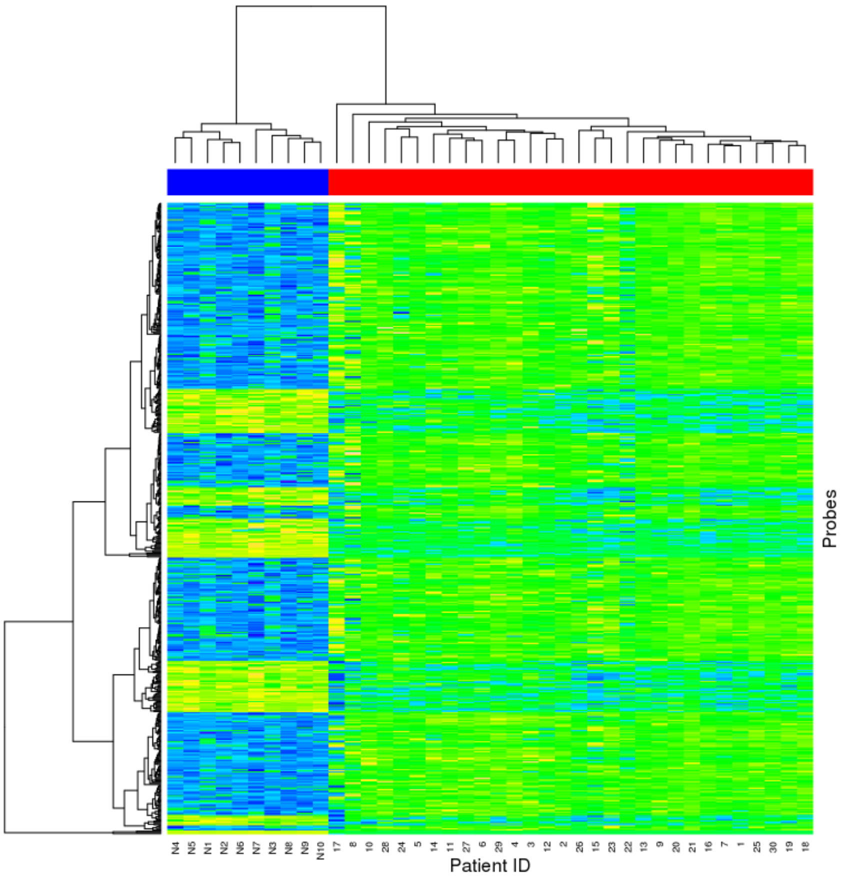

Figure 8: A heatmap comparing differential expression between cancer samples purified using *ISOpureR* (red columns) and normal samples (blue columns). In this case, 496 transcript levels are differentially expressed between cancer and normal, Holm-adjusted p-value of 0.01.

[4] Bhattacharjee, A. and Richards, W. G. and Staunton, J. and Li, C. and Monti, S. and Vasa, P. and Ladd, C. and Beheshti, J. and Bueno, R. and Gillette, M. and Loda, M. and Weber, G. and Mark, E. J. and Lander, E. S. and Wong, W. and Johnson, B. E. and Golub, T. R. and Sugarbaker, D. J. and Meyerson, M., Classification of human lung carcinomas by mRNA expression profiling reveals distinct adenocarcinoma subclasses. Proc. Natl. Acad. Sci. U.S.A. 98(24) 13790–13795 (2001) `http://www.pnas.org/content/98/24/13790.full`

[5] Gautier, L. and Cope, L. and Bolstad, B. M. and Irizarry, R. A., affy–analysis of Affymetrix GeneChip data at the probe level. Bioinformatics. 20(3), 307-315 (2004) `http://www.ncbi.nlm.nih.gov/pubmed/12118244`

[6] Beer, D.G., Kardia, S.L., Huang, C.C., Giordano, T.J., Levin, A.M., Misek, D.E., Lin, L., Chen, G., Gharib, T.G., Thomas, D.G., Lizyness, M.L., Kuick, R., Hayasaka, S., Taylor, J.M., Iannettoni, M.D., Orringer, M.B., Hanash, S., Gene-expression profiles predict survival of patients with lung adenocarcinoma. Nat. Med. 8(8), 816-824 (2002) `http://www.ncbi.nlm.nih.gov/pubmed/12118244`

[7] Ritchie, M.E., Phipson, B., Wu, D., Hu Y., Law C.W., Shi W. and Smyth G.K. limma powers differential expression analyses for RNA-sequencing and microarray studies. Nucleic Acids Research, 43 (2015) doi: 10.1093/nar/gkv007.

[8] Cock, P. Molecular Organisation and Assembly in Cells (Using R to draw a Heatmap from Microarray Data) `http://www2.warwick.ac.uk/fac/sci/moac/people/students/peter_cock/r/heatmap/` (accessed 2015-03-19)
