# Supplementary material for: ISOpureR: an R implementation of a computational purification algorithm of mixed tumour profiles
Source: BMC Bioinformatics. 2015 May 14;16:156. doi: 10.1186/s12859-015-0597-x (PMC4429941; doi:10.1186/s12859-015-0597-x)
Supplement: Additional file 3 — (Figure) Bayesian network model for the ISOpure statistical model. [file 12859_2015_597_MOESM3_ESM.pdf]

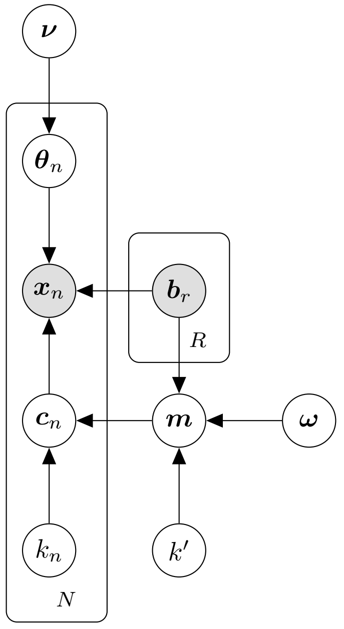

Figure 1 Bayesian network model of ISOpure. The graph represents the relationships between the variables in the ISOpure model, where the conditional probability of a given variable is only dependent on its parents in the graphs. The shaded circles, $\boldsymbol{x}_n$ and $\boldsymbol{b}_r$, represent the observed variables. The estimated model parameters (unshaded circles) are conditioned on the estimated model hyper-parameters (also unshaded). The N and R in the corners of the plates (rectangles) indicate that the variables inside are repeated N and R times, once for each patient and healthy profile, respectively. This figure is modified from [33] and appears identically in the *ISOpureR* vignette.
