# Supplementary material for: ISOpureR: an R implementation of a computational purification algorithm of mixed tumour profiles
Source: BMC Bioinformatics. 2015 May 14;16:156. doi: 10.1186/s12859-015-0597-x (PMC4429941; doi:10.1186/s12859-015-0597-x)
Supplement: Additional file 4 — (Figures) Bayesian network and flowchart diagrams for the cancer profile estimation and patient profile estimation steps of the ISOpure algorithm. [file 12859_2015_597_MOESM4_ESM.pdf]

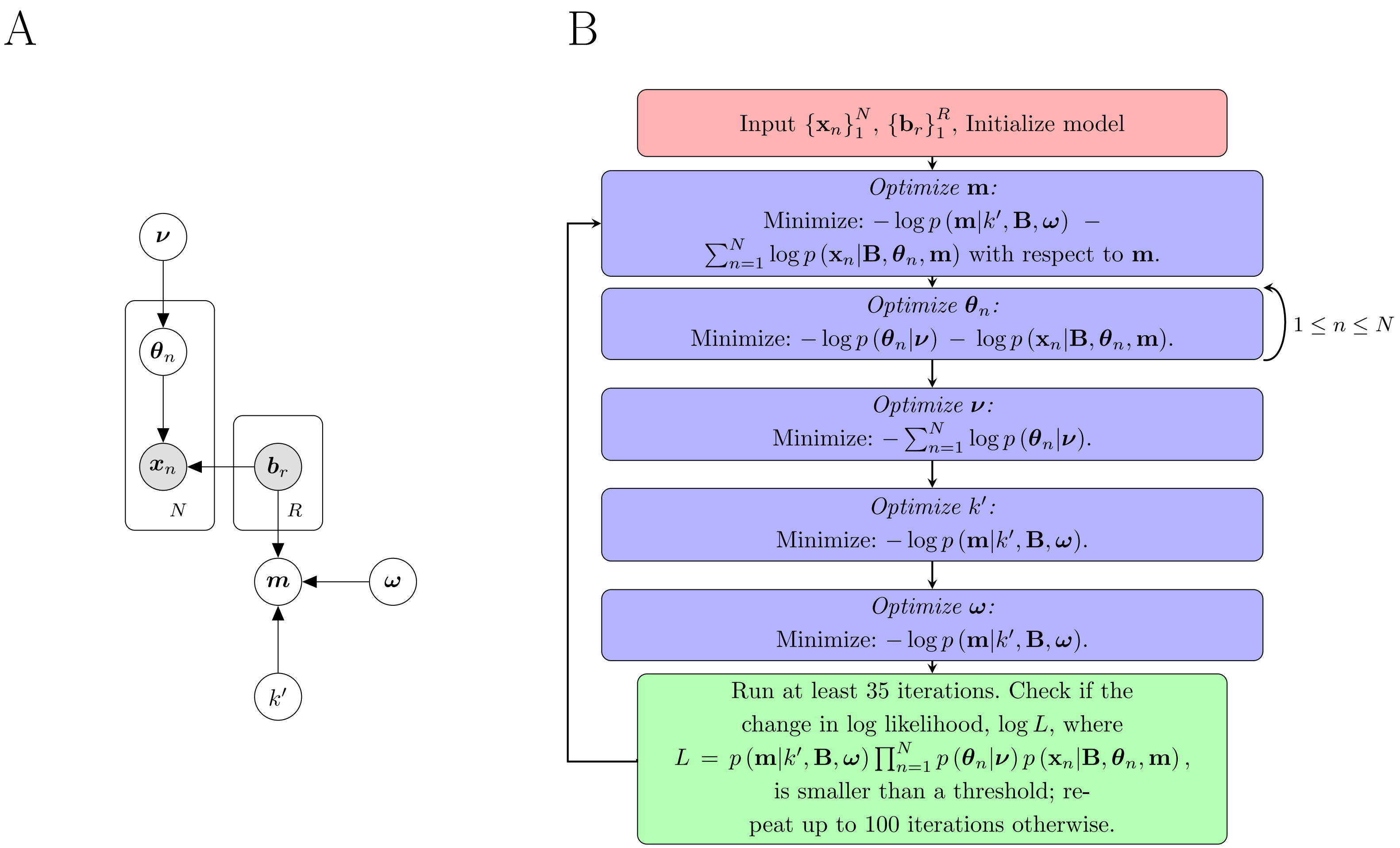

Figure 1 Cancer Profile Estimation (CPE) step. A diagram of the CPE first step of ISOpure. **(A)** The Bayesian network model for the CPE step. The inputs are the tumour profiles $\mathbf{x}_n$ and the normal profiles $\mathbf{b}_r$. The average cancer profile $\mathbf{m}$ and the cancer and healthy proportions $\boldsymbol{\theta}_n$ are estimated in this step. The $\boldsymbol{\nu}$, $\boldsymbol{\omega}$ and k' represent hyper-parameters in the hierarchical model. **(B)** Flowchart illustrating the algorithm for the CPE step. At each step, the Polak-Ribière conjugate gradient method with Wolfe-Powell stopping criteria is used to estimate variables of the same type. Each variable is estimated once during each iteration.

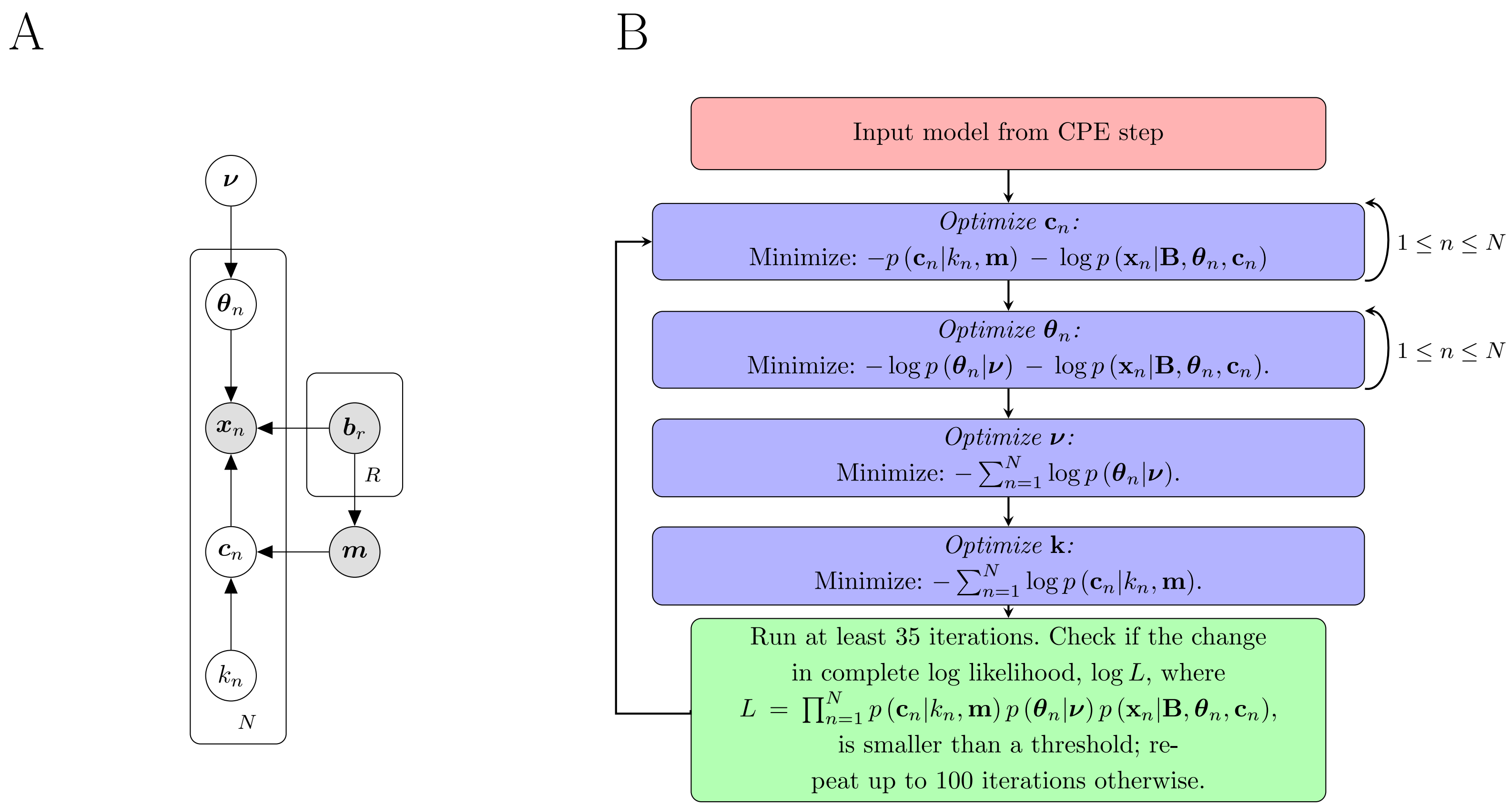

Figure 2 Patient Profile Estimation (PPE) step. A diagram of the PPE second step of ISOpure. **(A)** The Bayesian network model for the PPE step. The inputs are the tumour profiles $\mathbf{x}_n$ and the normal profiles $\mathbf{b}_r$; the average profile $\mathbf{m}$ is kept constant from the value obtained in the CPE step and α_n, the last entry of $\boldsymbol{\theta}_n$, also remains constant. The healthy proportions $\boldsymbol{\theta}_n$ and the patient-specific cancer profiles $\mathbf{c}_n$ are estimated in this step. The $\boldsymbol{\nu}$ and k_n represent hyper-parameters in the hierarchical model. **(B)** Flowchart illustrating the algorithm for the PPE step. At each step, the Polak-Ribière conjugate gradient method with Wolfe-Powell stopping criteria is used to estimate variables of the same type. Each variable is estimated once during each iteration.
