# Supplementary material for: ISOpureR: an R implementation of a computational purification algorithm of mixed tumour profiles
Source: BMC Bioinformatics. 2015 May 14;16:156. doi: 10.1186/s12859-015-0597-x (PMC4429941; doi:10.1186/s12859-015-0597-x)
Supplement: Additional file 5 — (Figures) A comparison of running time for different dataset sizes (different number of transcripts or different number of tumour samples). [file 12859_2015_597_MOESM5_ESM.pdf]

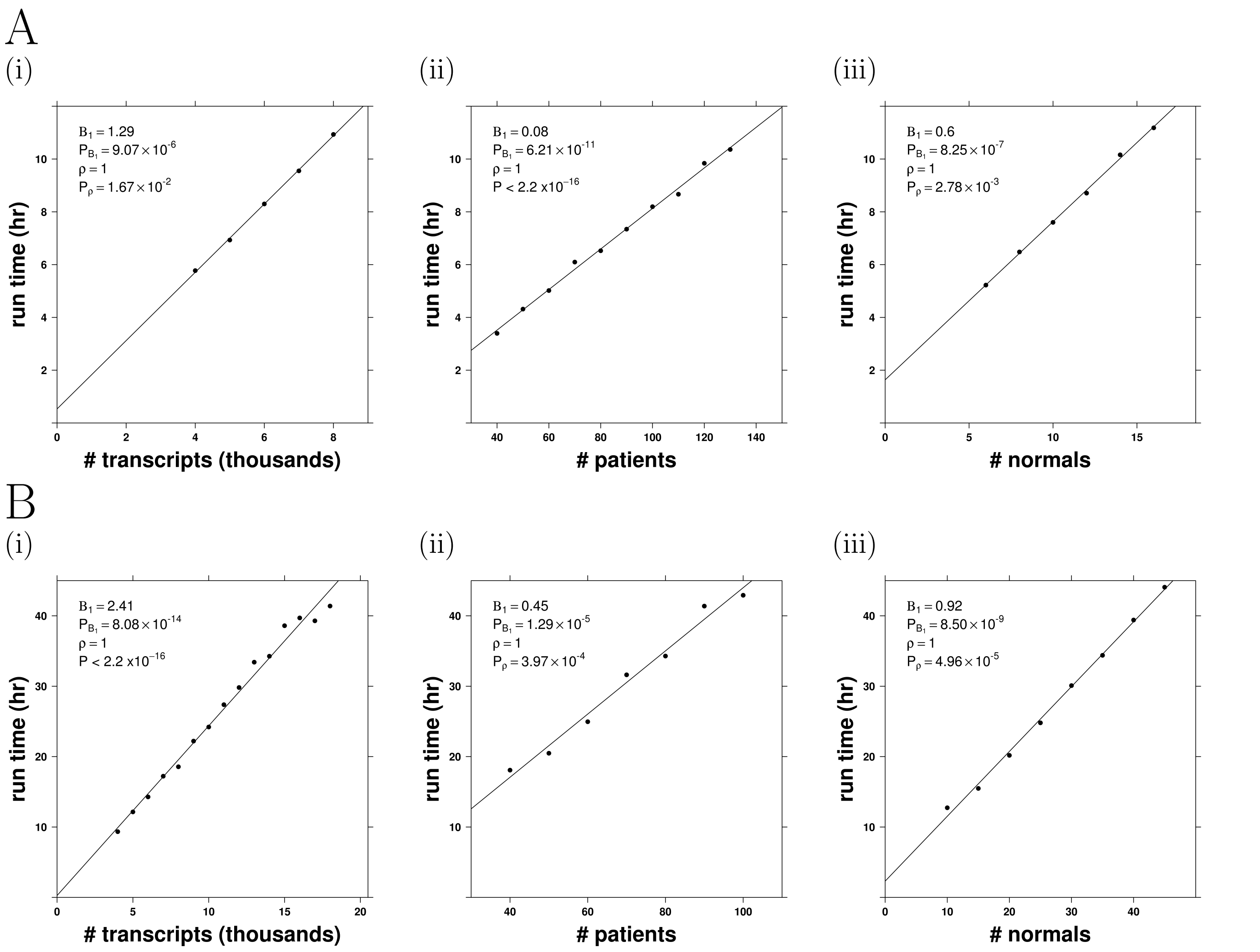

Figure 1 ***ISOpureR*** **run times for the (A) Bhattacharjee dataset and the (B) Wang dataset.** A comparison of run times for different number of transcripts, patients, and normals for the Bhattacharjee lung adenocarcinoma dataset and the Wang prostate cancer dataset. The full Bhattachajee dataset contains 8383 transcripts, 139 patients, and 17 normal samples and the full Wang dataset contains 18185 transcripts, 109 patients, and 45 normal samples. For **(i)** the number of patients and normals remained the same as for the full datasets, and only the number of transcripts was changed. For **(ii)** only the number of patients was changed, and for **(iii)** only the number of normal samples was changed.
