# Supplementary material for: ISOpureR: an R implementation of a computational purification algorithm of mixed tumour profiles
Source: BMC Bioinformatics. 2015 May 14;16:156. doi: 10.1186/s12859-015-0597-x (PMC4429941; doi:10.1186/s12859-015-0597-x)
Supplement: Additional file 6 — (Figures) A comparison of parameters estimated by the MATLAB and R implementations of ISOpure for the Bhattacharjee, Wallace, and Wang datasets. [file 12859_2015_597_MOESM6_ESM.pdf]

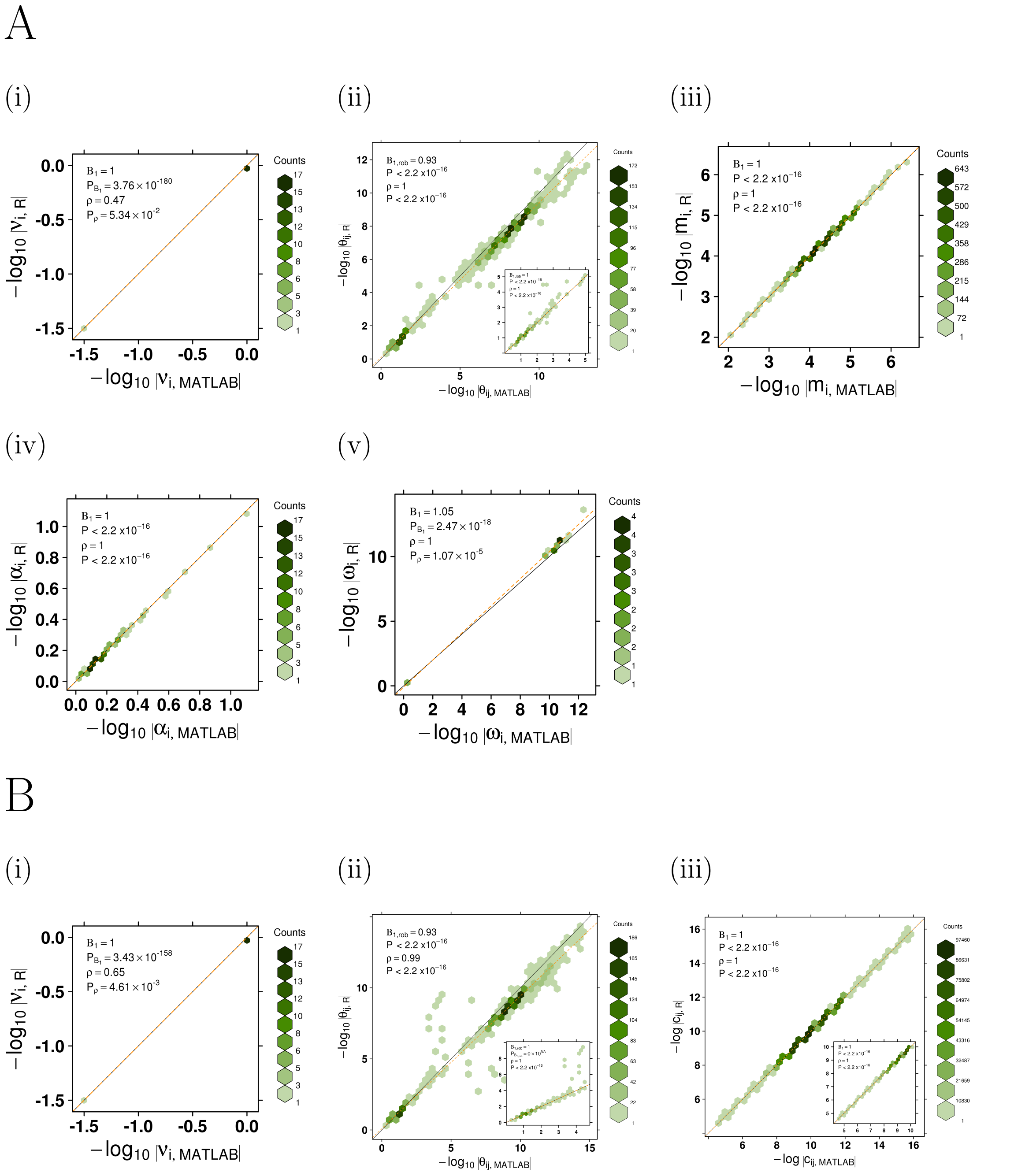

Figure 1 A comparison of parameters estimated by the MATLAB and R implementations of ISOpure for the Bhattacharjee dataset. Each plot shows the entries of a parameter estimated using *ISOpureR* plotted against the corresponding entries estimated using the MATLAB code. The parameter is an average over 50 models run with different initial conditions. The line $y = x$ is indicated in black, and the linear regression line, or robust regression line for $\boldsymbol{\theta}$, is dashed orange. $\mathbf{(A)}$ Parameters from the Cancer Profile Estimation step of ISOpure are: (i) $\boldsymbol{\nu}$, the hyper-parameter for the Dirichlet distribution over $\boldsymbol{\theta}$, (ii) $\boldsymbol{\theta}$, the proportion of a patient sample from a known healthy-tissue profile, (iii) $\mathbf{m}$, the average mRNA abundance cancer profile, (iv) α, the fraction of cancer cells for every patient sample, (v) $\boldsymbol{\omega}$ a hyper-parameter for the Dirichlet distribution over $\mathbf{m}$. $\mathbf{(B)}$ Parameters from the Patient Profile Estimation step of ISOpure are (i) $\boldsymbol{\nu}$, the hyper-parameter for the Dirichlet distribution over $\boldsymbol{\theta}$, (ii) $\boldsymbol{\theta}$, the proportion of a patient sample from a known healthy-tissue profile, (iii) $\mathbf{c}_n$, the purified mRNA abundance cancer profile for each patient.

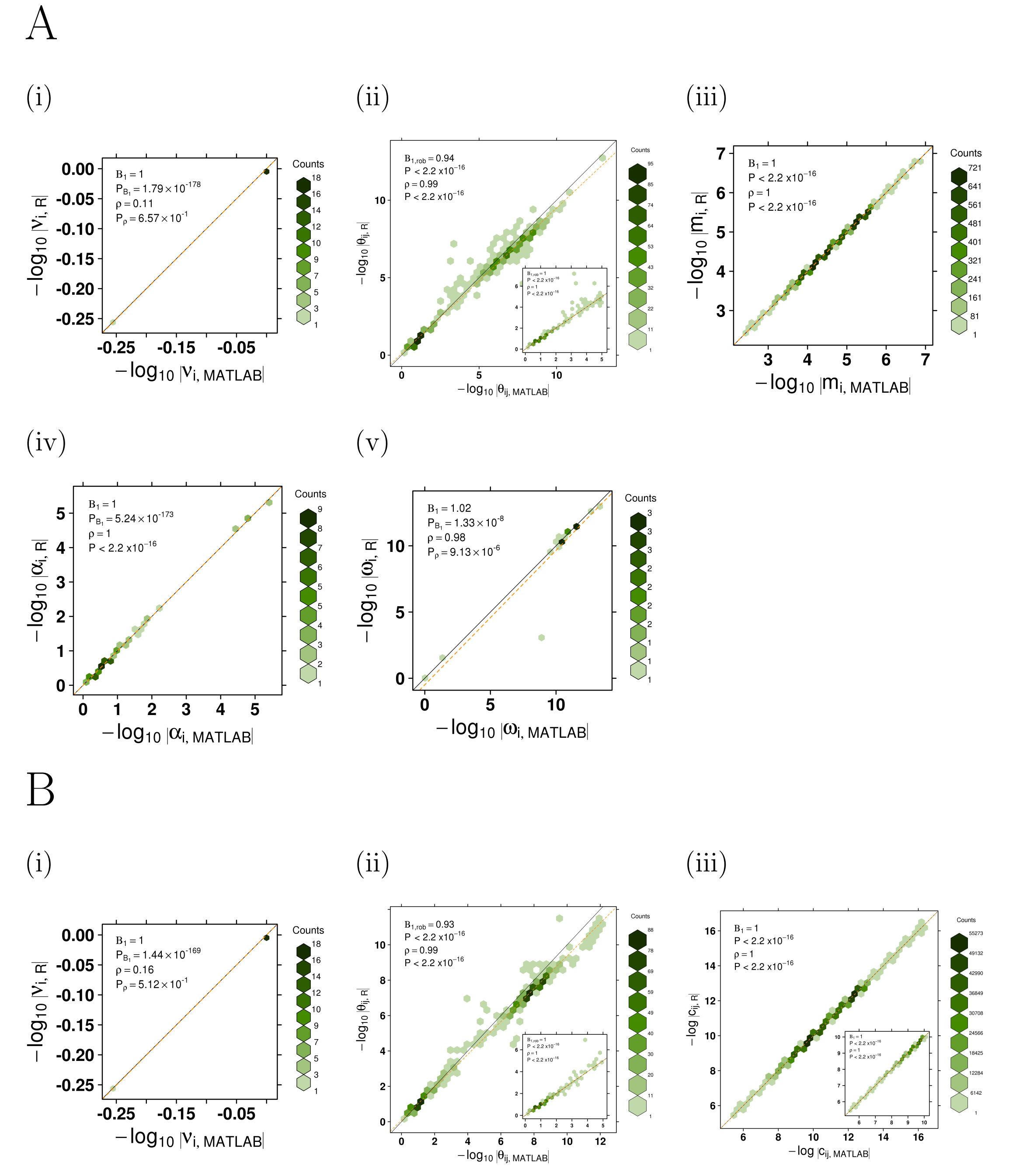

Figure 2 A comparison of parameters estimated by the MATLAB and R implementations of ISOpure for the Wallace dataset. Each plot shows the entries of a parameter estimated using *ISOpureR* plotted against the corresponding entries estimated using the MATLAB code. The parameter is an average over 50 models run with different initial conditions. The line $y = x$ is indicated in black, and the linear regression line, or robust regression line for $\boldsymbol{\theta}$, is dashed orange. A description of the parameters from the Cancer Profile Estimation step $(\mathbf{A})$ and the Patient Profile Estimation step $(\mathbf{B})$ of ISOpure is given in Figure 1.

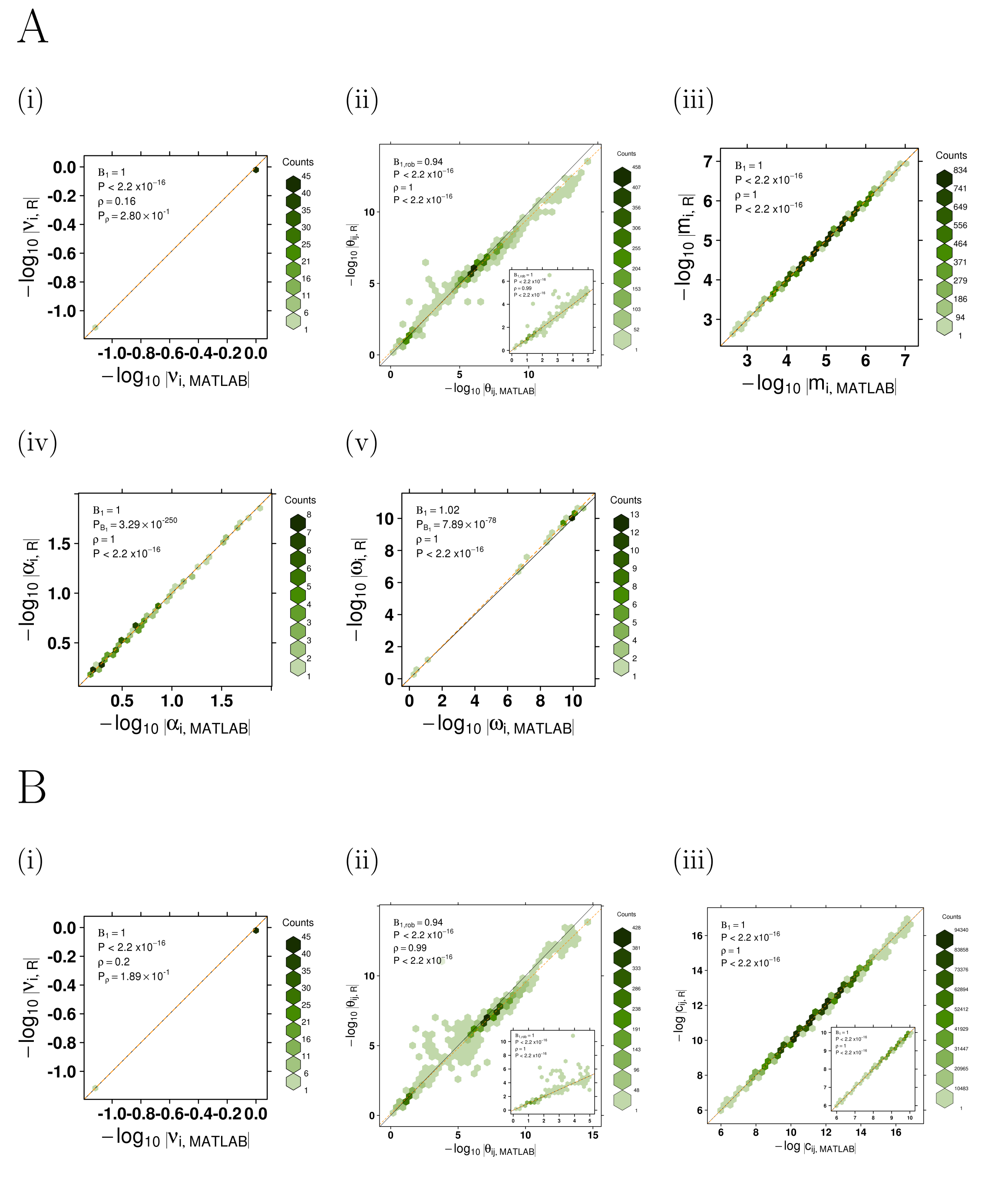

Figure 3 A comparison of parameters estimated by the MATLAB and R implementations of ISOpure for the Wang dataset. Each plot shows the entries of a parameter estimated using *ISOpureR* plotted against the corresponding entries estimated using the MATLAB code. The parameter is an average over 25 MATLAB models run with different initial conditions and 13 R models; models converging to a local minimum were omitted. The line $y = x$ is indicated in black, and the linear regression line, or robust regression line for $\boldsymbol{\theta}$, is dashed orange. A description of the parameters from the Cancer Profile Estimation step $(\mathbf{A})$ and the Patient Profile Estimation step $(\mathbf{B})$ of ISOpure is given in Figure 1.
