# Supplementary material for: ISOpureR: an R implementation of a computational purification algorithm of mixed tumour profiles
Source: BMC Bioinformatics. 2015 May 14;16:156. doi: 10.1186/s12859-015-0597-x (PMC4429941; doi:10.1186/s12859-015-0597-x)
Supplement: Additional file 7 — (Tables) Statistics for differences in parameters estimated by the MATLAB and R implementations of ISOpure for the Beer, Bhattacharjee, Wallace, and Wang datasets. [file 12859_2015_597_MOESM7_ESM.pdf]

Table 1 **Statistics for the MATLAB and R parameter means taken over 49 models for the Beer lung adenocarcinoma dataset with different initial conditions, random restarts omitted. The initial conditions are initial values of ν (or `vv` in the code), which were uploaded from a saved file, so that both MATLAB and R code was run first with initial ν_1, then ν_2, up to ν_{50}. One model was omitted (number 14) as the MATLAB algorithms gave a θ value that was zero. Each row corresponds to a single parameter. For some parameters (e.g. 'omega' and 'omega-larger') a comparison is made only among the larger components of the vector or matrix parameter, so that only components that are larger than 10^{-5} are compared. The number after the parameter name indicates whether the parameter is estimated in Step 1 (CPE) or Step 2 (PPE) of ISOpure.**

Parameter-step	Fraction (of vector/matrix entries) more than 0.05 different	Check bias (difference $(\bar{x}_M - \bar{x}_R)$ centered about 0?)				Summary of fractional difference $\lvert\bar{x}_M - \bar{x}_R\rvert / \lvert\bar{x}_M\rvert$					
		Median difference	Mean difference	Min value of entry in $\bar{x}_M$	Max value of entry in $\bar{x}_M$	Min	1st Qu	Median	Mean	3rd Qu	Max
vv-1	0	-6.1016E-12	-0.000244289	1	17.0155521605	1.217E-13	1.644E-12	6.102E-12	0.00001436	1.283E-11	0.0001579
omega-1	0.6	3.0407E-11	-5.2514E-18	8.0486E-11	0.3452114569	0.003724	0.01843	0.08632	0.2216	0.3488	0.9432
omega-larger-1	0	-0.00085601	-9.3184E-9	0.1586577679	0.3452114569	0.003724	0.00594	0.01204	0.01233	0.01843	0.02151
mm-1	0	1.5441E-9	-7.8246E-20	1.9958E-7	0.0049742972	4.446E-9	0.00001683	0.00002946	0.00008061	0.0000471	0.03653
alphapurities-1	0	-4.8267E-5	-0.000037519	0.2613000309	0.896986884	5.266E-7	0.00006131	0.00007417	0.00009067	0.00009506	0.001065
kappa-2	0	-2479.6695	-2479.66945	5013768.8169	5013768.8169	0.0004946	0.0004946	0.0004946	0.0004946	0.0004946	0.0004946
vv-2	0	-2.52889E-11	-0.0002443	1	17.0155521883	3.076E-12	1.495E-11	2.529E-11	0.00001436	9.22E-11	0.0001579
theta-1	0.6860465116	-1.1342E-8	3.75190E-6	3.0763E-32	0.5694067422	7.042E-7	0.00189	1.603	6.152	2.917	2115
theta-larger-1	0.0561403509	3.6889E-6	1.2197E-5	1.1299E-5	0.5694067422	7.042E-7	0.0002018	0.0005109	0.0264	0.001777	1.147
theta-2	0.6872093023	-7.6443E-10	3.7519E-6	5.4128E-16	0.5648821074	5.469E-7	0.0044	2.098	4.34	3.38	210.1
theta-larger-2	0.1003344482	9.28076E-6	1.0836E-5	3.9365E-5	0.5648821074	5.469E-7	0.000263	0.000891	0.03324	0.006765	1
log_cc-2	0	1.2994E-5	-0.00001765	-16.3363	-5.2260	2.473E-11	0.000002116	0.000004737	0.00001647	0.00001076	0.01575

Table 2 **Statistics for the MATLAB and R parameter means taken over 50 models for the Bhattacharjee lung adenocarcinoma dataset with different initial conditions, random restarts omitted. Table entries are as described for Table 1.**

Parameter-step	Fraction (of vector/matrix entries) more than 0.05 different	Check bias (difference $(\bar{x}_M - \bar{x}_R)$ centered about 0?)				Summary of fractional difference $\lvert\bar{x}_M - \bar{x}_R\rvert/\lvert\bar{x}_M\rvert$					
		Median difference	Mean difference	Min value of entry in $\bar{x}_M$	Max value of entry in $\bar{x}_M$	Min	1st Qu	Median	Mean	3rd Qu	Max
vv-1	0	2.0264E-12	0.0002117	1	31.714359445	3.744E-13	1.256E-12	2.782E-12	0.000006674	6.69E-12	0.0001201
omega-1	0.8823529412	1.2534E-11	-2.7139E-18	4.5876E-13	0.5632330201	0.00008124	0.5172	0.5254	0.49	0.5293	0.9706
omega-larger-1	0	-1.8996E-10	-1.8996E-10	0.4367669792	0.5632330201	0.00008124	0.00008712	0.000093	0.000093	0.00009888	0.0001048
mm-1	0	-9.3252E-11	5.6262E-19	4.1112E-7	0.0088030088	1.17E-8	0.000009127	0.00002018	0.00004189	0.00004047	0.01024
alphapurities-1	0	1.4901E-5	0.000029914	0.0789636302	0.9585213184	4.793E-8	0.00001336	0.00002493	0.00005345	0.00004363	0.002601
kappa-2	0	-27688.1445	-27688.1445	2445477.6228	2445477.6228	0.01132	0.01132	0.01132	0.01132	0.01132	0.01132
vv-2	0	-5.06297E-11	0.0002116697	1	31.7143593985	3.285E-12	2.524E-11	8.455E-11	0.000006674	1.748E-10	0.0001201
theta-1	0.7735928904	-1.1067E-8	-1.7596E-6	1.5581E-13	0.4743590714	5.744E-7	0.2345	1.574	1.882	2.35	368.9
theta-larger-1	0.0463458111	-9.9138E-6	-6.8066E-6	1.3322E-5	0.4743590714	5.744E-7	0.0001196	0.0004059	0.02257	0.001186	2.218
theta-2	0.7850190436	-8.1726E-10	-1.7596E-6	2.7643E-15	0.487851479	0.000007179	0.24	1.947	8545	2.806	19730000
theta-larger-2	0.1630615641	-2.2472E-6	-2.4209E-6	3.3047E-5	0.487851479	0.000007179	0.0005785	0.002211	0.04925	0.01539	1.444
log_cc-2	0	-0.000173596	-0.000144165	-15.8492	-4.59372272	2.309E-11	0.00001615	0.00003454	0.00004863	0.00006113	0.009799

Table 3 Statistics for the MATLAB and R parameter means taken over 50 models for the Wallace prostate cancer dataset with different initial conditions, random restarts omitted. Table entries are as described for Table 1.

Parameter-step	Fraction (of vector/matrix entries) more than 0.05 different	Check bias (difference ($\bar{x}_M - \bar{x}_R$) centered about 0?)				Summary of fractional difference $\lvert\bar{x}_M - \bar{x}_R\rvert/\lvert\bar{x}_M\rvert$					
		Median difference	Mean difference	Min value of entry in $\bar{x}_M$	Max value of entry in $\bar{x}_M$	Min	1st Qu	Median	Mean	3rd Qu	Max
vv-1	0	-2.4849E-12	-0.000162842	1	1.8006873086	4.146E-13	1.137E-12	3.84E-12	0.00009043	7.165E-12	0.001718
omega-1	0.8333333333	-7.2499E-13	5.6714E-18	4.6268E-14	0.9537608364	0.0009908	0.2534	0.2935	27820	0.3034	500700
omega-larger-1	0	0.0003176316	0.0003176316	0.0462391618	0.9537608364	0.0009908	0.009287	0.01758	0.01758	0.02588	0.03418
mm-1	0.000247117	2.1561E-9	3.0594E-19	1.3356E-7	0.0037100411	7.715E-9	0.0003915	0.0008654	0.00167	0.001629	0.08743
alphapurities-1	0	-0.000590367	-0.000925895	3.8943E-6	0.8085713126	0.0001313	0.002196	0.003371	0.005969	0.00568	0.04826
kappa-2	0	-105785.0196	-105785.0196	7492434.9443	7492434.9443	0.01412	0.01412	0.01412	0.01412	0.01412	0.01412
vv-2	0	-6.6549E-12	-0.000162842	1	1.8006873177	1.149E-12	9.154E-12	1.188E-11	0.00009043	2.093E-11	0.001718
theta-1	0.6425120773	-4.9353E-8	5.14386E-5	1.0829E-13	0.6840541574	2.25E-8	0.005756	0.4344	1.824	1.539	962.4
theta-larger-1	0.1643286573	-5.3594E-7	0.000128392	1.0016E-5	0.6840541574	2.25E-8	0.000572	0.002832	0.07588	0.01807	5.145
theta-2	0.6674718196	-2.2519E-8	5.1439E-5	7.3369E-13	0.6839767863	2.875E-8	0.00511	1.23	45 4	2.195	551000
theta-larger-2	0.1084598698	1.02264E-8	0.0001407151	1.1949E-5	0.6839767863	2.875E-8	0.0003422	0.00196	0.04691	0.008074	3.222
log_cc-2	0	-5.1722E-6	-0.000735235	-16.3646	-5.45440504	8.626E-11	0.00003801	0.00008475	0.0001689	0.0001677	0.01827

Table 4 **Statistics for the parameter means taken over 25 MATLAB models and 13 R models for the Wang prostate cancer dataset with different initial conditions, random restarts omitted. Table entries are as described for Table 1.**

Parameter-step	Fraction (of vector/matrix entries) more than 0.05 different	Check bias (difference $(\bar{x}_M - \bar{x}_R)$ centered about 0?)				Summary of fractional difference $\lvert\bar{x}_M - \bar{x}_R\rvert / \lvert\bar{x}_M\rvert$					
		Median difference	Mean difference	Min value of entry in $\bar{x}_M$	Max value of entry in $\bar{x}_M$	Min	1st Qu	Median	Mean	3rd Qu	Max
vv-1	0	9.6823E-13	0.000071652	1	13.0794596305	3.464E-14	8.706E-13	3.213E-12	0.000005478	7.867E-12	0.000252
omega-1	0.9333333333	6.1459E-11	-5.8291E-19	2.4123E-11	0.5460422952	0.002445	0.3972	0.4065	0.3683	0.4103	0.5841
omega-larger-1	0	-0.00091896	-2.2161E-8	0.0781153089	0.5460422952	0.002445	0.003057	0.00367	0.006668	0.008779	0.01389
mm-1	0.000439923	-4.7380E-10	3.5550E-19	9.4286E-8	0.0023426968	2.299E-7	0.0003454	0.0007949	0.002002	0.001814	0.1269
alphapurities-1	0	-6.6136E-5	-6.8464E-5	0.0130102198	0.6586440473	0.00001167	0.0002833	0.0006972	0.00147	0.001196	0.04372
kappa-2	0	100983.6743	100983.6743	10644669.8099	10644669.8099	0.009487	0.009487	0.009487	0.009487	0.009487	0.009487
vv-2	0	2.9102E-11	7.1652E-5	1	13.079459593	1.338E-12	2.821E-11	5.212E-11	0.000005478	1.185E-10	0.000252
theta-1	0.7060142712	-3.5299E-8	1.5214E-6	5.4516E-15	0.6577503907	3.039E-7	0.03265	0.2094	1.184	0.985	1133
theta-larger-1	0.293598234	3.2333E-7	6.1511E-6	1.0008E-5	0.6577503907	3.039E-7	0.001354	0.008085	0.09757	0.08842	3.588
theta-2	0.8046890928	-1.9771E-8	1.5214E-6	2.4630E-15	0.6583594005	2.603E-7	0.1085	0.787	2.361	1.707	2541
theta-larger-2	0.1804922516	1.2199E-5	1.2365E-5	1.0486E-5	0.6583594005	2.603E-7	0.001047	0.004165	0.2148	0.02157	134.4
log_cc-2	0	2.7827E-6	-0.00102363	-16.8257	-5.983422661	1.863E-11	0.00003867	0.00008644	0.0001851	0.0001813	0.03398
