# Supplementary figures and images for: ISOpureR: an R implementation of a computational purification algorithm of mixed tumour profiles
Source: BMC Bioinformatics. 2015 May 14;16:156. doi: 10.1186/s12859-015-0597-x (PMC4429941; doi:10.1186/s12859-015-0597-x)

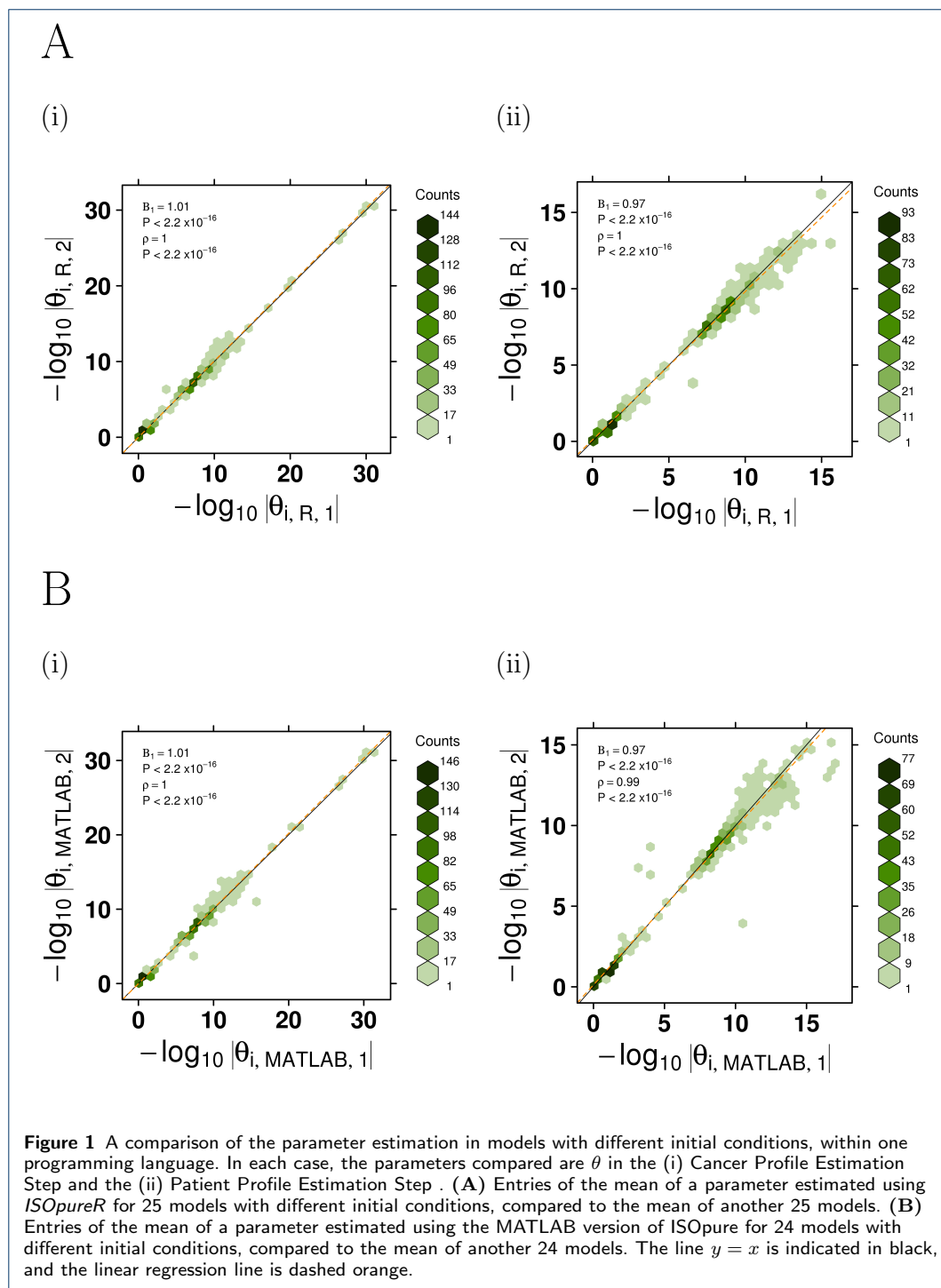

Supplement: Additional file 8 — (Figure) A comparison of the parameter estimation in models with different initial conditions, one programming language. [file 12859_2015_597_MOESM8_ESM.pdf]
