# Supplementary material for: ISOpureR: an R implementation of a computational purification algorithm of mixed tumour profiles
Source: BMC Bioinformatics. 2015 May 14;16:156. doi: 10.1186/s12859-015-0597-x (PMC4429941; doi:10.1186/s12859-015-0597-x)
Supplement: Additional file 9 — Recommendations for translating code from MATLAB to R. [file 12859_2015_597_MOESM9_ESM.pdf]

# Recommendations for translating code from MATLAB to R

## General

1. Organize files according to the R package structure from the beginning. MATLAB code can have a complex directory hierarchy, whereas R packages do not. Disaggregating this hierarchy is a technically simple and essential first step; attempting to do it later is very time consuming. The minimum suggestion (even if a package is not immediately created) is to collect all functions in one directory with no sub-directories and to create corresponding .Rd files in another directory.

2. Arithmetic operations with integer or double data-types should be straightforward to implement, as both MATLAB and R align in large part with the IEEE 754 standard according to the online documentation (Section on "Avoiding Common Problems with Floating-Point Arithmetic" at `http://www.mathworks.com/help/matlab/matlab_prog/floating-point-numbers.html`, and Section C.8 of the "R Installation and Administration" manual at `http://www.r-project.org/`, where IEEE 754 is also denoted IEC 60559; both accessed 11/11/2014). However, operations with complex numbers, comparisons, and data type changes (especially automatic changes in R affecting the matrix data type) will be problematic. Some examples are included in the next section.

3. Numerical accuracy is more important than speed at first. Attempts to speed-up or optimize code can introduce numerical differences that are very difficult to pin down.

4. It is useful to eliminate randomness, for instance by loading values for initial conditions from a saved file for both MATLAB and R. Once code has no random functions, changing the random seed should not affect output in any way. There is no "numerical noise" – round-off, truncation, underflow and overflow errors within each language will be completely reproducible given different random seeds, but no random functions.

5. Use good coding practices. For instance, do not hard code pathnames in functions comparing MATLAB and R output data, and allow flexibility in the directories where the data is saved; to keep data and results organized, a new directory will need to be created for each test.

## Code-level recommendations

1. Basic operators and functions are different in MATLAB and R on non-integer/double types or on "non-standard" input. Therefore, it is necessary to know if any intermediate operations result in `NaN` or `NA`, in complex numbers, in negative numbers input to a `log`, etc.

   (a) **(Greater/less than)** Comparisons with `NaN` values in MATLAB return False (*i.e.* a value of 0), whereas in R they return `NA`. Including such comparisons in conditional statements will make R stop in an error, but not MATLAB. (There is also a difference in comparisons of complex values, but in the complex value case R produces an error.)

✓

MATLAB:
```
>> 4 < 5

ans =
       1
```

R:
```
> 4 < 5

[1] TRUE
```

✗

```
>> NaN < 5

ans =
     0
```

```
> NaN < 5

[1] NA
```

| | MATLAB | R |
|---|---|---|
| ✗ | `>> 3+2i < 5`<br><br>`ans =`<br>`1`<br><br>`>> 3+2i < 2`<br><br>`ans =`<br>`0` | `> complex(real=3, imaginary=2) < 5`<br><br>`Error in`<br>`complex(real = 3, imaginary = 2) < 5 :`<br>`invalid comparison with complex values` |

(b) **(Transpose)** The transpose of a matrix with complex values in MATLAB also includes taking the conjugate.

✓

MATLAB:

```
>> A = [1, 1+1*i; 2, 4]

A =

     1.0000 1.0000 + 1.0000i
     2.0000 4.0000
```

R:

```
> A <- matrix(
+ c(1,2,complex(real=1,imaginary=1),4),
+ 2,
+ 2)

> A
      [,1] [,2]
[1,] 1+0i 1+1i
[2,] 2+0i 4+0i
```

✗

```
>> A'

ans =

    1.0000              2.0000
    1.0000 - 1.0000i    4.0000
```

```
> t(A)

      [,1] [,2]
[1,] 1+0i 2+0i
[2,] 1+1i 4+0i
```

(c) **(Logarithm)** The `log` of a negative number returns a complex number in MATLAB, and `NaN` in R. The analogue of the `log` function in R is the MATLAB `reallog`.

✓

MATLAB:

```
>> log(3)

ans =
    1.0986
```

R:

```
> log(3)

[1] 1.098612
```

✗

```
>> log(-3)

ans =
   1.0986 + 3.1416i
```

```
> log(-3)

[1] NaN
Warning message:
In log(-3) : NaNs produced
```

2. **(Data types)** MATLAB and R have different functions to check if a number is real, complex, etc. but similar function names may not have the same output.

✗

MATLAB:

```
>> isnumeric(3+7i)

ans =
     1
```

R:

```
> is.numeric(complex(real=3,imaginary=7))

[1] FALSE
```

3. **(Matrices)** When taking out a single row or column from a matrix, in R the row will be changed into a vector type. To avoid this, use the `drop=FALSE` option.

| | MATLAB: | R: |
|---|---|---|
| ✓ | `>> A = [1, 3, 5; 2, 4, 6]`<br><br>`A =`<br><br>`    1     3     5`<br>`    2     4     6` | `> A <- matrix(c(1,2,3,4,5,6),2,3)`<br><br>`> A`<br>`     [,1] [,2] [,3]`<br>`[1,]    1    3    5`<br>`[2,]    2    4    6` |
| ✗ | `>> B = A(:,2)`<br><br>`B =`<br><br>`     3`<br>`     4` | `> B <- A[,2]`<br><br>`> B`<br>`[1] 3 4`<br><br>`> str(B)`<br>` num [1:2] 3 4` |
| ✓ | `>> B = A(:,2)`<br><br>`B =`<br><br>`     3`<br>`     4` | `> B <- A[,2, drop=FALSE]`<br><br>`> B`<br>`     [,1]`<br>`[1,]    3`<br>`[2,]    4` |

4. **(Matrices)** Use `as.matrix()` to convert dataframes to a matrix, but use `matrix()` to convert a vector into a row matrix; using `as.matrix()` on a vector will automatically make it a one-column matrix.

```
> v <- 1:5;

> as.matrix(v, nrow=1, ncol=5)
     [,1]
[1,]    1
[2,]    2
[3,]    3
[4,]    4
[5,]    5

> matrix(v, nrow=1, ncol=5)
     [,1] [,2] [,3] [,4] [,5]
[1,]    1    2    3    4    5
```

5. **(Precision)** Check the computing precision of any function used, especially if it is faster! For instance, `max.col(M)` applied to a matrix `M` has a relative tolerance of 1e-5 relative to the largest entry in a row, and breaks ties at random.

```
> M <- matrix(c(10, 2, 5, 9, 3, 6), 3, 2);

> M
     [,1] [,2]
[1,]   10    9
[2,]    2    3
[3,]    5    6

> max.col.indices <- max.col(M)
> max.col.indices
[1] 1 2 2
```

```
> M <- matrix(c(10.000007, 3.0000005, 5, 10.000002, 3.0000008, 6), 3, 2)

> M
          [,1]      [,2]
[1,] 10.000007 10.000002
[2,]  3.000001  3.000001
[3,]  5.000000  6.000000

> max.col.indices <- max.col(M)
> max.col.indices
[1] 1 1 2

> max.col.indices <- max.col(M)
> max.col.indices
[1] 2 2 2
```

## Debugging in R

1. One of the most useful debugging tools in R is `options(error=recover)` which opens in browser mode at the occurrence of an error. When debugging a complicated, iterative function, it is very useful not to stop at every iteration of a troublesome function, or print pages of intermediate numbers, but be able to stop only at the location of the error.

## Acknowledgments

Thanks to Lawrence Heisler, Gerald Quon and Andre Masella for their R help.
